# On the Uniqueness of Schwarzschild–de Sitter Spacetime

**DOI:** 10.1007/s00205-023-01860-1

**Published:** 2023-03-09

**Authors:** Stefano Borghini, Piotr T. Chruściel, Lorenzo Mazzieri

**Affiliations:** 1 Università degli Studi di Milano-Bicocca, Via Roberto Cozzi 55, 20125 Milan, MI Italy; 2 University of Vienna, Gravitational Physics Boltzmanngasse 5, 1090 Vienna, Austria; 3 Università degli Studi di Trento, via Sommarive 14, 38123 Povo, TN Italy

## Abstract

We establish a new uniqueness theorem for the three dimensional Schwarzschild–de Sitter metrics. For this, some new or improved tools are developed. These include a reverse Łojasiewicz inequality, which holds in a neighborhood of the extremal points of any smooth function. We further prove the smoothness of the set of maxima of the lapse, whenever this set contains a topological hypersurface. This leads to a new strategy for the classification of well behaved static solutions of vacuum Einstein equations with a positive cosmological constant, based on the geometry of the maximum-set of the lapse.

## Introduction

A basic and fundamental question in the study of the mathematical aspects of General Relativity is the classification of the static solutions to the Einstein equations, starting from the case of vacuum solutions. The first result in this field is the celebrated Israel’s Theorem [30], concerning the uniqueness of the Schwarzschild solution, among the asymptotically flat static solutions to the vacuum Einstein equations. Different proofs and refinements of Israel’s result have been proposed by many authors [3, 14, 17, 31, 32, 48, 49, 52].

A similar analysis has been performed for asymptotically hyperbolic static solutions. Wang [51] and the second author together with Herzlich [19] proved a uniqueness theorem for the Anti de Sitter solution (compare [13]), whereas Lee and Neves [41] obtained a similar result for the Kottler spacetimes with negative mass aspect (compare [26, Remark 3.4]).

In contrast with the significant amount of achievements in the case $\Lambda \leqq 0$, very little is known when $\Lambda$ is positive, except in the locally conformally flat case [40], for perturbations of the model solutions [28] and under pinching assumptions on the curvature [4].

In order to set the stage for the problem, we recall that a $(n+1)$–dimensional vacuum static spacetime is a Lorentzian manifold $(X,\gamma )$ having the form

$$\begin{aligned} X={\mathbb {R}}\times M,\quad \gamma =-u^2 dt^2+g, \end{aligned}$$

where $t\in {\mathbb {R}}$, (*M*, *g*) is a *n*-dimensional Riemannian manifold with boundary and $u:M\rightarrow {\mathbb {R}}$ is a nonnegative smooth function vanishing precisely on the boundary of *M*, and satisfying the vacuum Einstein equations

$$\begin{aligned} \textrm{Ric}(\gamma )\,=\,\frac{2\Lambda }{n-1}\gamma . \end{aligned}$$

It is clear that a static vacuum spacetime is uniquely identified by the triple (*M*, *g*, *u*), which will then be referred to as a *static triple* or *static solution*. The function *u* is usually called *lapse function* or *static potential* in the literature. The connected components of $\partial M$ are referred to as *horizons*, as they typically correspond to Killing horizons in the associated spacetime.

Since we are interested in the positive cosmological constant case, it is natural to set $\Lambda =n(n-1)/2$, and work under such a normalization, being clear that the general case is readily recovered through a simple rescaling. With this normalization, the most important family of *n*-dimensional static triples with positive cosmological constant, the so-called Birmingham-Kottler (BK) solutions (see [7, 34]), can be described as

1.1 $$\begin{aligned} g = \frac{dr^2}{u^2} + r^2 g_\Sigma , \quad u^2 = 1 -r^2 - \frac{2m}{r^{n-2}} , \end{aligned}$$

where *m* is a real constant and $(\Sigma ,g_\Sigma )$ is a $(n-1)$-dimensional simply connected Riemannian manifold with

1.2 $$\begin{aligned} \textrm{Ric}(g_\Sigma ) = (n-2) g_\Sigma . \end{aligned}$$

In our terminology, the Schwarzschild-de Sitter family represents a special case of BK solutions, in which the Riemannian manifold $(\Sigma ,g_\Sigma )$ is taken to be a $(n-1)$-dimensional sphere with the canonical metric.

Observe that, in order to get a static triple out of formula (1.1), it is mandatory for the lapse function *u* to be well defined. It is clear that this happens only if the quantity $1-r^2-2mr^{2-n}$ is nonnegative for some positive values of *r*. This restricts the choice of the parameter *m* to the interval $0\leqq m<m_{\textrm{max}}$, where $m_{\textrm{max}}$ is a constant only depending on *n* (see (5.1) for the explicit expression). All in all, we have that for any $0< m<m_{\textrm{max}}$, the BK solutions (1.1) provide well defined static triples in the region $\{r_-(m)<r<r_+(m) \}$, where $0<r_-(m)<r_+(m)$ are the two positive roots of $1-r^2-2mr^{2-n}$. Also, recall that the Nariai solutions (see [47]) can be defined as the limit of the BK solutions, when $m\rightarrow m_{\textrm{max}}$. An explicit expression for these is given by

$$\begin{aligned} g = \frac{1}{n} dr^2 + \frac{n-2}{n} g_\Sigma ,\quad u=\sin (r), \end{aligned}$$

where again $g_\Sigma$ is a Riemannian metric on $\Sigma$ satisfying (1.2).

Having the above families of model solutions in mind, the concept of *virtual mass* was introduced in [11, 12], through a comparison algorithm (see also [6, 20] for similar ideas). Building on this concept, we prove the following uniqueness result for the Schwarzschild–de Sitter black hole:

### Theorem 1.1

(Uniqueness of the Schwarzschild–de Sitter Black Hole) Let (*M*, *g*, *u*) be a compact orientable 3-dimensional static triple with cosmological constant $\Lambda >0$, where (*M*, *g*) is a smooth Riemannian manifold with boundary and $u \in {\mathscr {C}}^\infty (M)$ is a nonnegative lapse function, whose zero set corresponds to $\partial M$. Assume that the set

$$\begin{aligned} \textrm{MAX}(u) \,=\,\{ x\in M:\,u(x)\,=\, u_\textrm{max}\} \end{aligned}$$

is disconnecting the manifold *M* into an outer region $M_+$ and an inner region $M_-$ having the same *virtual mass* $0< m < 1/(3\sqrt{3})$. Then (*M*, *g*, *u*) is isometric to the Schwarzschild–de Sitter solution with mass parameter *m*.

For a better understanding of the above statement, we need to recall some more terminology and concepts, still given in the spirit of the comparison with the BK solutions. Depending upon the value assumed by the *(normalized) surface gravity* $|\nabla u|/u_{\textrm{max}}$, each horizon is either of cosmological type (low surface gravity) or of black hole type (high surface gravity). When the set on which *u* attains its maximum separates *M* into two components $M_\pm$, it was shown in [11, 12] how to assign a *virtual mass* $m_\pm$ to each component; see Section 5 for more details. The virtual masses are shown there to obey the inequality $m_+\geqq m_-$, provided $M_+$ is bounded only by horizons of cosmological type (we then say that $M_+$ is an *outer region*). In agreement with (5.1) and by definition, for $n=3$, the whole range of the virtual mass parameter *m* is $0\leqq m \leqq 1/(3\sqrt{3})$. The case $m=0$ leads to the de Sitter space [11, Theorem 2.3]. The case $m =1/(3\sqrt{3})$ leads to the Nariai solution. The known explicit solutions have the property that the virtual masses coincide, i.e., $m_+ =m= m_-$, for some $0< m < 1/(3\sqrt{3})$. It was shown in [12, Theorem 1.9] that, in three space-dimensions, equality of the masses and connectedness of the part of the horizon bounding $M_+$ implies that the sharp area bound (see [12, Theorem 1.4])

$$\begin{aligned} |\partial M_+| \, \leqq \, 4\pi r_+^2(m) \end{aligned}$$

is saturated and thus (*M*, *g*, *u*) arises from the Schwarzschild-de Sitter solution. Theorem 1.1 can be seen as an improvement of this result: indeed, we are able to remove the hypothesis on the connectedness of the cosmological horizon. Perhaps more significantly, a completely new strategy of proof is used.

As sketched above, the central idea in [12] was in a sense closer to the concepts developed in [3], as it consisted in establishing sharp *a priori* bounds relating the area of the horizons to the virtual mass of the corresponding region (Riemannian Penrose-type inequalities), characterizing the equality case in terms of the model solutions, and then showing that in some physically relevant situation (connected cosmological horizon, same mass for the outer and the inner region) the equality was actually achieved in the a priori bounds. At the heart of the new strategy is instead a detailed analysis of the locus $\textrm{MAX} (u)$, established in a fairly large generality and which we believe of independent interest. Forever, before describing the most significant achievements of this analysis, let us discuss the plausibility of our assumptions, making some comments about what it is worth to expect from the level set $\textrm{MAX} (u)$. On the one hand, it can be argued that containing a co-dimension 1 stratum that disconnects the underlying manifold is a highly non-generic hypothesis, even for a real analytic function like the lapse. On the other hand, it would be fair to compare our assumptions with the ones that are commonly accepted and employed to prove the classical Uniqueness Theorem for the Schwarzschild Black Hole, when $\Lambda = 0$. In this case, it is standard to assume that the metric is *asymptotically flat* and that the lapse function $u_0$ is attaining its supremum at spatial infinity (see for example [14, Assumption 1]). Building on these two conditions, it is not difficult to realise that the blow-down limit of the large level sets of $u_0$ consists of a totally geodesic round sphere. Clearly, such a blow-down limit is the object that should be compared with $\textrm{MAX}(u)$ in the present framework. Hence, from this point of view, it might have been natural to impose a spherical geometry on $\textrm{MAX}(u)$, whereas in our Theorem 1.1 we are not even assuming that it is a smooth manifold. Analogous considerations also apply to Theorem 1.2 below, where we will suppose that $\textrm{MAX}(u)$ is containing a totally umbilic component, whose induced metric obeys the Einstein equation (1.2). There is therefore a sense in which our assumptions are weaker than the ones of the classical Israel’s Theorem.

Let us then describe in more detail our analysis of $\textrm{MAX}(u)$. Our first main result in this context is a *reverse Łojasiewicz inequality*, Theorem 2.2 below, which provides an estimate on the gradient of a smooth function in terms of its increment near and away from its maximum set. This is an improved version of [12, Proposition 2.3] (see also [10, Section 1.1.5]). The result is used to control potential singularities in the function *W* which appears in the proof the gradient estimates (5.3) below and in turns plays a key role in the proof of Theorem 1.1. Our next main result is Corollary 3.4, which shows that the top stratum (defined in (3.1) below) of the set of maxima of an analytic function with Laplacian bounded away from zero is a smooth embedded submanifold. This result, which sheds significant new light on the structure of static solutions, is crucial for our strategy, as it allows us to invoke the uniqueness part of the Cauchy–Kovalewskaya Theorem to classify the solutions, leading to

### Theorem 1.2

(Geometric characterization of BK and Nariai solutions) Let (*M*, *g*, *u*) be a compact orientable *n*-dimensional, $n\geqq 3$, static triple with cosmological constant $\Lambda >0$. Suppose that the top stratum of $\textrm{MAX}(u)$ is not empty and denote by $\Sigma$ one of its connected components. Then $\Sigma$ is a compact smooth hypersurface without boundary. Furthermore, if the metric induced on $\Sigma$ by *g* is Einstein and $\Sigma$ is totally umbilic, then the universal cover of (*M*, *g*) is isometric to either a Birmingham-Kottler or a Nariai solution.

Some comments might be in order concerning the claims in [45], seemingly rendering our Theorem 1.1 obsolete. The claims in [45] are based on calculational mistakes, which can be seen as follows: in order to justify their assertion the authors get rid of the boundary by doubling the manifold. Note that applying this to the Schwarzschild-de Sitter space metric produces a manifold with topology $S^{1}\times S^2$. A conformal blow-up of the metric at a point using the Green function is then asserted to lead to an asymptotically flat metric with non-negative scalar curvature and zero mass. If this were correct, one could use the rigidity part of the positive energy theorem to conclude that the doubled manifold is diffeomorphic to a sphere $S^3$, which is clearly not the case for $S^{1}\times S^2$.

### Summary

The rest of this paper is structured as follows: In Section 2 we will prove the reverse Łojasiewicz inequality (Theorem 2.1). In Section 3 we will analyze the regularity properties of the extremal set of an analytic function with controlled Laplacian. More precisely, in Theorem 3.1 we show how to expand a function in a neighborhood of the set of the maximum (or minimum) points. Building on this, under the hypothesis that the Hessian does not vanish, we show in Theorem 3.3 and Corollary 3.4 that the $(n-1)$-dimensional part of the extremal level set is a real analytic hypersurface without boundary. It is worth remarking that the results in Sects. 2 and 3 are not exclusive of the static realm, but they hold more generally for large classes of real-valued functions. In fact, other recent applications of these same properties have appeared in [5], where they have been used to study critical metrics of the volume functional, and in [2] where the classical torsion problem is discussed. From Section 4 we start focusing exclusively on static solutions. We show that the estimates given by Theorem 3.3 allow to trigger the Cauchy-Kovalevskaya Theorem 4.1, leading to a proof of Theorem 1.2 (see Theorem 4.2). Section 5 is devoted for the most part to the statement and proof of Theorem 5.1, which is a quite general result stating that if an outer region is next to an inner region, then the virtual mass of the outer region is necessarily greater than or equal to the one of the inner region. This theorem is supplemented by a rigidity statement in the case of equality of the virtual masses. In dimension 3, it is possible to combine this rigidity statement with Theorem 1.2, leading to the proof of Theorem 1.1. Finally, in Section 6 we discuss how to exploit the Cauchy-Kovalevskaya scheme proposed in Section 4 in order to produce local static solutions.

## Reverse Łojasiewicz Inequality

Let (*M*, *g*) denote a smooth *n*-dimensional Riemannian manifold, possibly with boundary, $n\geqq 2$. Given a smooth function $f:M\rightarrow {\mathbb {R}}$, we will denote by $f_{\textrm{max}}$ the maximum value of *f*, when achieved, and by

$$\begin{aligned} \textrm{MAX}(f)\,=\,\{x\in M:\,f(x)\,=\,f_{\textrm{max}}\} \end{aligned}$$

the set of the maxima of *f*, when nonempty. We will assume that $\textrm{MAX}(f)$ does not meet the boundary of *M*, if there is one.

We start by recalling the following classical result by Łojasiewicz, concerning the behaviour of an analytic function near a critical point:

### Theorem 2.1

(Łojasiewicz inequality [42, Théorème 4], [38]) Let (*M*, *g*) be a real analytic Riemannian manifold and let $f:M\rightarrow {\mathbb {R}}$ be an analytic function. Then for every point $p\in M$ there exists a neighborhood $U_p\ni p$ and real numbers $c_p>0$ and $1\leqq \theta _p<2$ such that for every $x\in U_p$ it holds

2.1 $$\begin{aligned} |\nabla f|^2(x)\,\geqq \,c_p\left| f(x)-f(p)\right| ^{\theta _p}. \end{aligned}$$

Let us make some comments on this result. First, we observe that the above theorem is only relevant when *p* is a critical point, as otherwise the proof is trivial. Another observation is that one can always set $c_p=1$ in (2.1), at the cost of increasing the value of $\theta _p$ and restricting the neighborhood $U_p$. Nevertheless, the inequality is usually stated as in (2.1), because one often wants to choose the optimal $\theta _p$. Let us also notice, in particular, that the above result implies the inequality

$$\begin{aligned} |\nabla f|(x)\,\geqq \,\left| f(x)-f(p)\right| \end{aligned}$$

in a neighborhood of any point of our manifold.

The gradient estimate (2.1) has found important applications in the study of gradient flows, as it allows to control the behaviour of the flow near the critical points. The validity of the Łojasiewicz Inequality has been extended to semicontinuous subanalytic functions in [8], and a generalized version of (2.1) has been developed by Kurdyka [37] for larger classes of functions. An infinite-dimensional version of (2.1) has been proved by Simon [50], who used it to study the asymptotic behaviour of parabolic equations near critical points. A Łojasiewicz-like inequality for noncompact hypersurfaces has been discussed in [22], where it is exploited as the main technical tool to prove the uniqueness of blow-ups of the mean curvature flow. We refer the reader to [9, 21, 25] and the references therein for a thorough discussion of various versions of the Łojasiewicz–Simon inequality, as well as for its applications.

On the other hand, to the authors’ knowledge, the opposite inequality has not been discussed yet. In this section we prove an analogous estimate from above of the gradient near the critical points. Before stating the result, let us make a preliminary observation. Suppose that we are given a Riemannian manifold (*M*, *g*) and a function $f\in {\mathscr {C}}^\infty (M)$. Let $p\in M$ be a critical point of *f*. If we restrict *f* to a curve $\gamma$ such that $\gamma (0)=p$ and ${\dot{\gamma }}(0)=X$, for some unit vector field $X\in T_p M$, we have

$$\begin{aligned} f\circ \gamma (t)\,=\,f(p)\,+\,\frac{\nabla ^2f(X,X)}{2}\,t^2\,+o(t^2), \end{aligned}$$

from which we compute that

2.2 $$\begin{aligned} \frac{\left( \frac{\partial }{\partial t} (f\circ \gamma )_{|_{t=\tau }}\right) ^2}{f(p)-f\circ \gamma (\tau )}\,=\,-\,2\,\frac{\big (\nabla ^2f(X,X)\big )^2\,\tau ^2\,+\,o(\tau ^2)}{\nabla ^2f(X,X)\,\tau ^2\,+\,o(\tau ^2)}. \end{aligned}$$

In particular, under the assumption that $\nabla ^2f(X,X)\ne 0$ at *p*, we have that the left hand side of (2.2) is locally bounded. As a consequence, we immediately obtain that the inequality

$$\begin{aligned} {|\nabla f|^2}\,\,\leqq \,\,c\,|f(p)-f| \end{aligned}$$

holds in a neighborhood of *p* for some constant $c>0$, provided we assume that $\nabla ^2f(p)(X,X)\ne 0$ for every $X\in T_p M$. The next theorem tells us that a slightly weaker bound is in force at the maximum (or minimum) points of *f*, without any assumptions on the Hessian.

### Theorem 2.2

(Reverse Łojasiewicz Inequality) Let (*M*, *g*) be a Riemannian manifold, let $f:M\rightarrow {\mathbb {R}}$ be a smooth function and let $\Sigma$ be a connected component of $\textrm{MAX}(f)$. If $\Sigma$ is compact, then for every $\theta <1$ and for any connected open neighborhood $\Omega \supset \Sigma$ with ${{\overline{\Omega }}}\cap \textrm{MAX}(f)=\Sigma$, there exists a real number $c>0$ such that for every $x\in \Omega$ it holds that

$$\begin{aligned} |\nabla f|^2(x)\,\leqq \,c\left[ f_{\textrm{max}}-f(x)\right] ^{\theta }. \end{aligned}$$

### Proof

The proof follows closely the one given in [12, Proposition 2.3], where the same inequality was proven for functions *f* satisfying (4.1). Let us start by defining the function

$$\begin{aligned} w\,=\,|\nabla f|^2-c\,(f_{\textrm{max}}-f)^\theta , \end{aligned}$$

where $c>0$ is a constant that will be chosen conveniently later. We compute

$$\begin{aligned} \nabla w\,=\,\nabla |\nabla f|^2\,+\,c\,\theta \, (f_{\textrm{max}}-f)^{-(1-\theta )}\,\nabla f, \end{aligned}$$

and taking the divergence of the above formula

$$\begin{aligned} \Delta w\,&=\,\Delta |\nabla f|^2\,+\,c\,\theta \, \frac{\Delta f}{(f_{\textrm{max}}-f)^{1-\theta }}\,+\,c\,\theta \,(1-\theta )\, \frac{|\nabla f|^2}{(f_{\textrm{max}}-f)^{2-\theta }}\\&=\,\Delta |\nabla f|^2\,+\,c\,\theta \, \frac{\Delta f}{(f_{\textrm{max}}-f)^{1-\theta }}\,+\,c\,\theta \,(1-\theta ) \,\frac{w}{(f_{\textrm{max}}-f)^{2-\theta }}\\ {}&\quad +\,c^2\theta \,(1-\theta )\, \frac{1}{(f_{\textrm{max}}-f)^{2-2\theta }}\,, \end{aligned}$$

where in the second equality we have used $|\nabla f|^2=w+c\,(f_{\textrm{max}}-f)^{\theta }$. We obtain the identity

2.3 $$\begin{aligned} \Delta w\,-\,c\,\theta \,(1-\theta )\,\frac{1}{(f_{\textrm{max}}-f)^{2-\theta }}\,w\,=\,\theta \, F\,\left[ \Delta f\,+\,(1-\theta )\,F\right] \,+\,\Delta |\nabla f|^2,\nonumber \\ \end{aligned}$$

where

$$\begin{aligned} F=\frac{c}{(f_{\textrm{max}}-f)^{1-\theta }}. \end{aligned}$$

Fix now a connected open neighborhood $\Omega$ of $\Sigma$ with smooth boundary $\partial \Omega$. Since $\Sigma$ is compact, we can suppose that ${\overline{\Omega }}$ is compact as well. By definition, we have

$$\begin{aligned} F\,\geqq \,\frac{c}{\max _{{\overline{\Omega }}}(f_{\textrm{max}}-f)^{1-\theta }},\quad \hbox {on } {\overline{\Omega }}. \end{aligned}$$

In particular, increasing the value of *c* if necessary, we can make *F* as large as desired. Since $\Delta f$ and $\Delta |\nabla f|^2$ are continuous and thus bounded in ${\overline{\Omega }}$, and since $0<\theta <1$, it follows that, for any *c* big enough, we have

$$\begin{aligned} \theta \, F\,[(1-\theta )\,F\,+\,\Delta f]\,+\,\Delta |\nabla f|^2\,\geqq \,0 \end{aligned}$$

on the whole ${\overline{\Omega }}$. For such values of *c*, the right hand side of (2.3) is nonnegative, that is,

2.4 $$\begin{aligned} \Delta w-\frac{\theta \,(1-\theta )\,c}{(f_{\textrm{max}}-f)^{2-\theta }}\,w\,\geqq \,0,\quad \hbox { in }\,{\overline{\Omega }}. \end{aligned}$$

Notice that the coefficient that multiplies *w* in (2.4) is negative, as $f\leqq f_{\textrm{max}}$ and $0<\theta <1$. Therefore, we can apply the Weak Maximum Principle [27, Corollary 3.2] to *w* in any open set where *w* is ${\mathscr {C}}^2$ – that is, on any open subset of $\Omega$ that does not intersect $\Sigma$. For this reason, it is convenient to choose a number $\varepsilon >0$ small enough so that the tubular neighborhood

$$\begin{aligned} B_\varepsilon (\Sigma )\,=\,\{x\in M:\,d(x,\Sigma )<\varepsilon \} \end{aligned}$$

is contained inside $\Omega$, and consider the set $\Omega _\varepsilon =\Omega \setminus \overline{B_\varepsilon (\Sigma )}$. Up to increasing the value of *c* if needed, we can suppose that

$$\begin{aligned} c\,\geqq \,\frac{\max _{\partial \Omega }|\nabla f|^2}{\min _{\partial \Omega }(f_{\textrm{max}}-f)^\theta }\,\geqq \,\max _{\partial \Omega }\frac{|\nabla f|^2}{(f_{\textrm{max}}-f)^\theta }, \end{aligned}$$

so that $w\leqq 0$ on $\partial \Omega$. Now we apply the Weak Maximum Principle to the function *w* on the open set $\Omega _\varepsilon$, obtaining

$$\begin{aligned} w\,\leqq \, \max _{\partial \Omega _\varepsilon } (w)\,=\,\max \left\{ \max _{\partial \Omega }(w),\,\max _{\partial B_\varepsilon (\Sigma )} (w)\right\} \,\leqq \,\max \left\{ 0,\,\max _{\partial B_\varepsilon (\Sigma )} (w)\right\} . \end{aligned}$$

Recalling the definition of *w*, taking the limit as $\varepsilon \rightarrow 0$, from the continuity of *f* and $|\nabla f|$, it follows that

$$\begin{aligned} \lim _{\varepsilon \rightarrow 0}\max _{\partial B_\varepsilon (\Sigma )} (w)\,=\,0, \end{aligned}$$

hence we obtain $w\leqq 0$ on $\Omega$. Translating *w* in terms of *f*, we have obtained that the inequality

$$\begin{aligned} |\nabla f|^2\,\leqq \, c \,(f_{\textrm{max}}-f)^\theta \end{aligned}$$

holds in $\Omega$, which is the neighborhood of $\Sigma$ that we were looking for. $\square$

In particular, we have the following simple refinement, that generalizes [12, Corollary 2.4]:

### Corollary 2.3

Under the hypotheses of Theorem 2.2, for any $p\in \Sigma$ and any $\alpha <1$, we have

$$\begin{aligned} \lim _{f(x)\ne f_{\textrm{max}},\ x\rightarrow p}\,\frac{|\nabla f|^2(x)}{[f_{\textrm{max}}-f(x)]^{\alpha }}\,=\,0. \end{aligned}$$

### Proof

From Theorem 2.2 it follows that we can choose constants $\alpha<\theta <1$ and $c>0$ such that

$$\begin{aligned} \frac{|\nabla f|^2}{[f_{\textrm{max}}-f]^\alpha }\,\leqq \, \frac{c\,[f_{\textrm{max}}-f]^\theta }{[f_{\textrm{max}}-f]^\alpha } \,=\, c \,[f_{\textrm{max}}-f]^{\theta -\alpha }, \end{aligned}$$

and, since we have chosen $\theta >\alpha$, the right hand side goes to zero as we approach *p*. This proves the claim. $\square$

## Regularity of the Extremal Level Sets

The well known Łojasiewicz Structure Theorem (established in [43], see also [36, Theorem 6.3.3]) states that the set of the critical points $\textrm{Crit}(f)$ of a real analytic function *f* is a (possibly disconnected) stratified analytic subvariety whose strata have dimensions between 0 and $n-1$. In particular, it follows that the set $\textrm{MAX}(f)\subseteq \textrm{Crit}(f)$ of the maxima of *f* can be decomposed as

3.1 $$\begin{aligned} \textrm{MAX}(f)\,=\,\Sigma ^0\sqcup \Sigma ^1\sqcup \dots \sqcup \Sigma ^{n-1}, \end{aligned}$$

where $\Sigma ^i$ is a finite union of *i*-dimensional real analytic submanifolds, for every $i=0,\dots ,n-1$. This means that, given a point $p\in \Sigma ^i$, there exists a neighborhood $p\in \Omega \subset M$ and a real analytic diffeomorphism $\phi :\Omega \rightarrow {\mathbb {R}}^n$ such that

$$\begin{aligned} \phi (\Omega \cap \Sigma ^i)\,=\,L\cap \phi (\Omega ), \end{aligned}$$

for some *i*-dimensional linear space $L\subset {\mathbb {R}}^n$. In particular, the set $\Sigma ^{n-1}$ is a real analytic hypersurface and is usually referred to as the *top stratum* of $\textrm{MAX}(f)$. In this section we show that we can get much more information about the behaviour of our function *f* around the maximum points that belong to the top stratum. The next theorem extends the result in [12, Proposition 2.9].

### Theorem 3.1

Let (*M*, *g*) be a real analytic Riemannian manifold, let $f:M\rightarrow {\mathbb {R}}$ be a real analytic function and let $p\in \textrm{MAX}(f)$ be a point in the top stratum of $\textrm{MAX}(f)$. Let $\Omega$ be a small neighborhood of *p* such that $\Sigma =\Omega \cap \textrm{MAX}(f)$ is contained in the top stratum and $\Omega \setminus \Sigma$ has two connected components $\Omega _+,\Omega _-$. We define the signed distance to $\Sigma$ as

$$\begin{aligned} r(x)\,=\, {\left\{ \begin{array}{ll} + \, d(x,\Sigma ), &{} \text { if } x\in {\overline{\Omega }}_+,\\ - \, d(x,\Sigma ), &{} \text { if } x\in {\overline{\Omega }}_-. \end{array}\right. } \end{aligned}$$

Then there is a real analytic chart $(r,\vartheta )=(r,\vartheta ^1,\dots ,\vartheta ^{n-1})$ with respect to which *f* admits the following expansion:

3.2 $$\begin{aligned} f(r,\vartheta )\,=\,f_{\textrm{max}}\,+\,\frac{\Delta f(0,\vartheta )}{2}\,r^2\,+r^3\,F(r,\vartheta ). \end{aligned}$$

Here *F* is a real analytic function. If we also assume that $\Delta f=-\varphi (f)$ for some real function $\varphi$, then

$$\begin{aligned}{} & {} f\,=\,f_{\textrm{max}}\,-\,\frac{\varphi (f_{\textrm{max}})}{2}\,r^2\,+\,\frac{\varphi (f_{\textrm{max}})}{6}\,\textrm{H}\,r^3\\{} & {} \,-\,\frac{\varphi (f_{\textrm{max}})}{24}\left[ |\textrm{h}|^2\,+\,2\,\textrm{H}^2\,+\,{\mathrm R}\,-\,{\mathrm R}^{\Sigma }\,-\,{\dot{\varphi }}(f_{\textrm{max}})\right] r^4\,+\,r^5\,G(r,\vartheta ), \end{aligned}$$

where *G* is a real analytic function. Here we have denoted by ${\dot{\varphi }}$ the derivative of $\varphi$ with respect to *f*, by $\textrm{H},\textrm{h}$ the mean curvature and second fundamental form of $\Sigma$ with respect to the normal pointing towards $\Omega _+$, and by ${\mathrm R},{\mathrm R}^\Sigma$ the scalar curvatures of *M* and $\Sigma$.

### Remark 3.2

We have formulated Theorem 3.1 in the context of real analytic geometry because of our intended application to the classification of static solutions of Einstein equations. It is, however, clear from the proof that the conclusions of Theorem 3.1 remain true for smooth functions on smooth Riemannian manifolds, with the following modifications: first, one should assume from the outset that $\Sigma$ is a smooth hypersurface (in which case the existence of a decomposition as in (3.1) becomes irrelevant); next, the functions *r*, *F* and *G* and the chart $(r,\vartheta )$ are smooth but not necessarily analytic.

### Proof

Let $(x^1,\dots ,x^n)$ be a chart centered at *p*, with respect to which the metric *g* and the function *f* are real analytic. From the fact that *p* belongs to the top stratum of $\textrm{MAX}(f)$, it follows that we can choose an open neighborhood $\Omega$ of *p* in *M*, where the signed distance *r*(*x*) is a well defined real analytic function (see for instance [35], where this result is discussed in full detail in the Euclidean space, however the proofs extend with small modifications to the Riemannian setting). More precisely, we have

$$\begin{aligned} r=\phi (x^1,\dots ,x^n), \end{aligned}$$

where $\phi$ is a real analytic function. Since *r* is a signed distance function, we have $|\nabla r|=1$, which implies in particular that one of the partial derivatives of $\phi$ has to be different from zero. Without loss of generality, let us suppose $\partial \phi /\partial x^1\ne 0$ in a small neighborhood $\Omega$ of *p*. As a consequence, we have that the function

$$\begin{aligned} U:\,{\mathbb {R}}^{n+1}\,\rightarrow \, {\mathbb {R}},\qquad U(r,x^1,\dots ,x^n)\,=\,r-\phi (x^1,\dots ,x^n). \end{aligned}$$

satisfies $\partial U/\partial x^1=-\partial \phi /\partial x^1\ne 0$ in $\Omega$. We can then apply the Real Analytic Implicit Function Theorem (see [36, Theorem 2.3.5]), from which it follows that there exists a real analytic function $u:{\mathbb {R}}^n\rightarrow {\mathbb {R}}$ such that

$$\begin{aligned} U(r,u(r,x^2,\dots ,x^n),x^2,\dots , x^n)=0. \end{aligned}$$

In other words, the change of coordinates from $(r,x^2,\dots ,x^n)$ to $(x^1,\dots ,x^n)$, which is obtained setting $x^1=u(r,x^2,\dots ,x^n)$, is real analytic. In particular *f* is a real analytic function also with respect to the chart $(r,\vartheta )$, where we have set $\vartheta =(\vartheta ^1,\dots ,\vartheta ^{n-1})$ with $\vartheta ^i=x^{i+1}$ for $i=1,\dots ,n-1$.

Since $\Sigma$ coincides with the points where $r=0$ inside $\Omega$, we can apply [44, Théorème 3.1] to get

$$\begin{aligned} f_{\textrm{max}}-f\,=\,r\,A, \end{aligned}$$

where $A=A(r,\vartheta )$ is a nonnegative real analytic function. For the reader convenience, we explicitly quote the invoked reference:

[44, Théorème 3.1]. *Let* $(x^1,\dots , x^m;y^1,\dots ,y^n)$ *be coordinates on an open set* $U\subseteq {\mathbb {R}}^{m+n}$. *Let* $f\in {\mathscr {C}}^\infty (U)$ *such that* $f(0,\dots ,0;y^1,\dots ,y^n)=0$. *Then there exist* $g_1,\dots , g_m\in {\mathscr {C}}^\infty (U)$ *such that* $f=\sum _{i=1}^m x^i\,g_i$.

Going on with the proof, we observe that the function $f_{\textrm{max}}-f$ achieves its minimum value 0 when $r=0$, thus

$$\begin{aligned} 0\,=\,\frac{\partial }{\partial r}(f_{\textrm{max}}-f)_{|_{r=0}}\,=\,\frac{\partial }{\partial r}(r\,A)_{|_{r=0}}\,=\,(A\,+\,r\,\partial A/\partial r)_{|_{r=0}}\,=\,A_{|_{r=0}}. \end{aligned}$$

In particular, we can apply [44, Théorème 3.1] again and we find that

3.3 $$\begin{aligned} f_{\textrm{max}}-f\,=\,r^2\,B, \end{aligned}$$

where $B=B(r,\vartheta )$ is a nonnegative real analytic function. Computing the Laplacian at the points where $r=0$, using the fact that the gradient of *f* vanishes there, we get that

$$\begin{aligned} \Delta f=g^{\alpha \beta }(\partial ^2_{\alpha \beta }f-\Gamma _{\alpha \beta }^\gamma \partial _\gamma f)\,=\,-2\,g^{rr}\,B\,=\,-2\,B \,. \end{aligned}$$

Applying again [44, Théorème 3.1], it follows that we can rewrite (3.3) as

3.4 $$\begin{aligned} f(r,\vartheta )\,=\,f_{\textrm{max}}\,+\,\frac{\Delta f(0,\vartheta )}{2}\,r^2\,+r^3\,F(r,\vartheta ). \end{aligned}$$

This concludes the first part of the proof. Notice that we could have obtained (3.4) faster by writing a priori the expansion $f=f_{\textrm{max}}+a(\vartheta )r+b(\vartheta )r^2+{\mathcal {O}}(r^3)$ and then using formula (3.5) below and the fact that $\partial f/\partial r=0$ on $\textrm{MAX}(f)$ to compute $a(\vartheta )=0$ and $\Delta f=2b(\vartheta )+{\mathcal {O}}(r)$. On the other hand, this alternative approach heavily relies on analyticity, whereas we want our proof to work in the smooth setting as well, in the spirit of Remark 3.2.

We now assume that $\Delta f=-\varphi (f)$ and we use this to gather more information on the real analytic function *F*. Set $\Sigma _\rho =\{r=\rho \}$ and observe that all $\Sigma _\rho$ with $\rho$ small enough are smooth, since $(r,\vartheta )=(r,\vartheta ^1,\dots ,\vartheta ^{n-1})$ is a real analytic chart and $|\nabla r|=1\ne 0$. In particular, of course, we have $\Sigma _0=\Sigma \cap \Omega$. On each $\Sigma _\rho$, the Laplacian $\Delta f$ of *f* satisfies the well-known formula

3.5 $$\begin{aligned} \Delta f\,=\,\nabla ^2f(\textrm{n},\textrm{n})\,+\,\textrm{H}\,\langle \nabla f\,|\,\textrm{n}\rangle \,+\,\Delta ^{\!\top } f, \end{aligned}$$

where $\textrm{n}=\partial /\partial r$ is the *g*-unit normal to $\Sigma _\rho$, $\textrm{H}$ is the mean curvature of $\Sigma _\rho$ with respect to $\textrm{n}$ and $\Delta ^{\!\top } f$ is the Laplacian of the restriction of *f* to $\Sigma _\rho$ with respect to the metric ${g{}^{\!\top }}$ induced by *g* on $\Sigma _\rho$. Evaluating  (3.5) at $\rho =0$, since $f=f_{\textrm{max}}$ and $|\nabla f|=0$ on $\Sigma _0$, we immediately get

$$\begin{aligned} \nabla ^2f(\nu ,\nu )\,=\,\Delta f, \end{aligned}$$

in agreement with the expansion (3.4).

Let us focus first on the quantity $\Delta ^{\!\top } f$. Calling ${g{}^{\!\top }}$ the metric induced by *g* on $\Sigma _\rho$ and $\Gamma ^\top$ the Christoffel symbols of ${g{}^{\!\top }}$, we have

$$\begin{aligned} \Delta ^{\!\top }f\,\,=\,\, ({g{}^{\!\top }})^{ij}\,\frac{\partial ^2 f}{\partial \vartheta ^i\partial \vartheta ^j}\,-\,({g{}^{\!\top }})^{ij}\,\,(\Gamma ^\top )^{k}_{ij}\,\,\frac{\partial f}{\partial \vartheta ^k}, \end{aligned}$$

where the indices *i*, *j*, *k* vary between 1 and $n-1$. On the other hand, if we assume that $\Delta f=-\varphi$ where $\varphi$ is a function of *f*, then from (3.4) we get

$$\begin{aligned} \frac{\partial ^2 f}{\partial \vartheta ^i\partial \vartheta ^j}_{|_{r=0}}= & {} \frac{\partial ^2 f}{\partial r\partial \vartheta ^i}_{|_{r=0}} \,=\, \frac{\partial ^3 f}{\partial r^2 \partial \vartheta ^i}_{|_{r=0}}\,=\, \frac{\partial ^3 f}{\partial r \partial \vartheta ^i \partial \vartheta ^j}_{|_{r=0}}\\ {}= & {} \frac{\partial ^4 f}{\partial r^2\partial \vartheta ^i\partial \vartheta ^j}_{|_{r=0}}\,=\,0, \end{aligned}$$

for all $i,j=1,\dots ,n-1$. From this, it easily follows that

$$\begin{aligned} \frac{\partial }{\partial r}\Delta ^{\!\top } f_{|_{r=0}}\,=\,\frac{\partial ^2}{\partial r^2}\Delta ^{\!\top }f_{|_{r=0}}\,=\,0. \end{aligned}$$

We now differentiate formula (3.5) twice with respect to *r*. Observing that $\Gamma _{rr}^r=0$ since $g_{rr}\equiv 1$, it is then easy to compute that the following identities hold on the set $\Sigma _0=\{r=0\}$:

$$\begin{aligned} \frac{\partial \Delta f}{\partial r}\,&=\,\frac{\partial ^3 f}{\partial r^3}\,+\,\textrm{H}\frac{\partial ^2 f}{\partial r^2}\,+\,\frac{\partial \textrm{H}}{\partial r}\,\frac{\partial f}{\partial r}\,,\\ \frac{\partial ^2 \Delta f}{\partial r^2}\,&=\,\frac{\partial ^4 f}{\partial r^4}\,+\,\textrm{H}\frac{\partial ^3 f}{\partial r^3}\,+\,2\,\frac{\partial \textrm{H}}{\partial r}\,\frac{\partial ^2 f}{\partial r^2}\,+\,\frac{\partial ^2 \textrm{H}}{\partial r^2}\,\frac{\partial f}{\partial r}\,. \end{aligned}$$

Since we also know that $\partial f/\partial r=0$ and $\partial ^2 f/\partial r^2=\Delta f=-\varphi (f_{\textrm{max}})$ on $\Sigma$, from the expansions above we deduce

$$\begin{aligned} \frac{\partial ^3 f}{\partial r^3}_{|_{r=0}}\,&=\,\varphi (f_{\textrm{max}})\,\textrm{H}\,.\\ \frac{\partial ^4 f}{\partial r^4}_{|_{r=0}}\,&=\,2\,\varphi (f_{\textrm{max}})\,\frac{\partial \textrm{H}}{\partial r}_{|_{r=0}}\,-\,\varphi (f_{\textrm{max}})\,\textrm{H}^2\,+\,\varphi (f_{\textrm{max}})\,{\dot{\varphi }}(f_{\textrm{max}})\,. \end{aligned}$$

Furthermore, the classical formula for the variation of the mean curvature (see for instance [29, Lemma 7.6]) gives

$$\begin{aligned} \frac{\partial \textrm{H}}{\partial r}_{|_{r=0}} \,=\,-\,|\textrm{h}|^2\,-\,\textrm{Ric}(\nu ,\nu )\,=\,\frac{1}{2}\left( {\mathrm R}^{\Sigma }\,-\,{\mathrm R}\,-\,|\textrm{h}|^2\,-\,\textrm{H}^2\right) , \end{aligned}$$

where in the latter equality we have used the Gauss Codazzi equation. Now that we have computed the third and fourth derivative of *f*, we can use this information to improve (3.4) and get the desired expansion of *f*. $\square$

Theorem 3.1 has some interesting consequences. Let us start from the simplest one. Given a point *p* in the top stratum of $\textrm{MAX}(f)$ and such that $\Delta f(p)\ne 0$, we can compute the explicit formula for the gradient of *f* as we approach *p* as

3.6 $$\begin{aligned} \lim _{x\not \in \textrm{MAX}(f),\,x\rightarrow p}\,\frac{|\nabla f|^2 (x)}{f_{\textrm{max}}-f(x)}\,&=\, \lim _{x\rightarrow p}\frac{(\Delta f(p))^2\,r^2(x)\,+\,{\mathcal {O}}(r^3(x))}{-({\Delta f}(p)/{2})\,r^2(x)\,+\,{\mathcal {O}}(r^3(x))}\, =\,-\,2\,\Delta f(p)\,. \end{aligned}$$

Notice in particular that formula (3.6) improves Corollary 2.3 for points in the top stratum of the set of the maxima having nonzero Laplacian.

Another useful consequence of Theorem 3.1 is the following regularity result on the top stratum of $\textrm{MAX}(f)$, that generalizes the result for static metrics proven in [12, Proposition 2.8] in order not to overburden the notation we will denote by $\Sigma$ the top stratum $\Sigma ^{n-1}$ in the decomposition (3.1) of $\textrm{MAX}(f)$:

### Theorem 3.3

Let (*M*, *g*) be a real analytic Riemannian manifold, let $f:M\rightarrow {\mathbb {R}}$ be a real analytic function and let $\Sigma$ be the top stratum of $\textrm{MAX}(f)$. If $p\in {\overline{\Sigma }}$ and $|\nabla ^2f|(p)\ne 0$, then $p\in \Sigma$.

### Proof

Let $p\in {\overline{\Sigma }}$ with $|\nabla ^2f|(p)\ne 0$, and let $\Omega$ be a small relatively compact open neighborhood of *p* in *M*. From what has been said it follows that we can choose $\Omega$ small enough so that

$$\begin{aligned} {\overline{\Sigma }}\cap \Omega \,=\,{\overline{\Sigma }}_1\cup \dots \cup {\overline{\Sigma }}_k \end{aligned}$$

for some $k\in {\mathbb {N}}$, where the $\Sigma _i$’s are connected real analytic hypersurfaces contained in the top stratum $\Sigma$ and $p\in {\overline{\Sigma }}_i$ for all $i=1,\dots ,k$.

From expansion (3.2), it follows that at any point $x\in \Sigma _i$, with respect to an orthonormal basis $\nu (x),X_1,\dots ,X_{n-1}$, where $\nu (x)$ is the unit normal to $\Sigma _i$, the Hessian of *f* is represented by a matrix of the form

$$\begin{aligned} \begin{bmatrix} \Delta f(x) &{} 0 &{} \cdots &{} 0 \\ 0 &{} 0 &{} \cdots &{} 0 \\ \vdots &{} \vdots &{} \ddots &{} \vdots \\ 0 &{} 0 &{} \cdots &{} 0 \end{bmatrix}\,. \end{aligned}$$

Since we are assuming that $|\nabla ^2f|\ne 0$ and that *x* is a maximum point for *f*, it is clear that $\Delta f(x)<0$. Using the fact that eigenvalues are continuous (see for instance [33, Chapter 2, Theorem 5.1]), it follows that the eigenvalues of the Hessian of *f* at *p* are $\Delta f(p)$ (which is negative by hypothesis), taken with multiplicity one, and 0, taken with multiplicity $n-1$. In particular, using again the continuity of the eigenvalues, restricting our neighborhood $\Omega$ if necessary, we can suppose that the minimal eigenvalue of $\nabla ^2f$ is simple on the whole $\Omega$. Since the function $\Omega \ni x\mapsto \nabla ^2f(x)$ is real analytic, it is known that the simple eigenvectors are real analytic in $\Omega$, see for instance the discussion in [33, Chapter 2, § 1] or in [39, Section 7], where a much more general statement in discussed. Therefore the vector $\nu$ extends to a real analytic unit-length vector field throughout $\Omega$. In particular, $\nu$ is real analytic on ${\overline{\Sigma }}\cap \Omega$ and the tangent space $T_p{\overline{\Sigma }}=\nu _p^{\perp }$ is well defined.

It is then easily shown (see for instance [15, p. 89, Lemma 1] or [1, Proposizione 3.7.2]) that there is an analytic chart $(x^1,\dots ,x^n)$ centered at *p* such that $\nu =\partial /\partial x^1$ in the whole $\Omega$. Since $\Sigma \cap \Omega$ is smooth and $\nu =\partial /\partial x^1$ is orthogonal to it, it follows that ${\overline{\Sigma }}\cap \Omega \subseteq \{x^1=0\}$. Since *f* is analytic and we have shown that $f_{\textrm{max}}-f=0$ on an hypersurface contained in $\{x^1=0\}$, it is immediate to deduce that it is possible to factor out $x^1$ from the Taylor expansion of $f_{\textrm{max}}-f$. It follows that $f_{\textrm{max}}-f=0$ on the whole $\{x^1=0\}$, which implies ${\overline{\Sigma }}\cap \Omega =\{x^1=0\}$. In particular, ${\overline{\Sigma }}$ is real analytic in a neighborhood of *p*.

To conclude that *p* is contained in the top stratum of $\textrm{MAX}(f)$, it remains to show that there are no other components of $\textrm{MAX}(f)$ that pass through *p*. In other words, it remains to show that, making $\Omega$ smaller if necessary, we have $\Omega \cap \textrm{MAX}(f)=\Omega \cap {\overline{\Sigma }}$. To this end, we first observe that, since we have already shown that ${\overline{\Sigma }}$ is a regular analytic hypersurface around *p*, the proof of Theorem 3.1 can be repeated without modifications to show that formula (3.2) holds in a neighborhood of *p*. More precisely, there exists a real analytic chart $(r,\vartheta )=(r,\vartheta ^1,\dots ,\vartheta ^{n-1})$ such that *r* is the signed distance from ${\overline{\Sigma }}$ and *f* has the following expansion in $\Omega$:

$$\begin{aligned} f(r,\vartheta )\,=\,f_{\textrm{max}}\,+\,\frac{\Delta f(0,\vartheta )}{2}\,r^2\,+r^3\,F(r,\vartheta ). \end{aligned}$$

Here *F* is real analytic. Setting $C=\sup _\Omega |F|$, we then have that in $\Omega$ it holds that

$$\begin{aligned} f(r,\vartheta )\,\leqq \,f_{\textrm{max}}\,+\,\frac{\Delta f(0,\vartheta )}{2}\,r^2\,+C|r|^3=\,f_{\textrm{max}}\,+\,r^2\left( \frac{\Delta f(0,\vartheta )}{2}\,+C|r|\right) . \end{aligned}$$

Since the points with $r=0$ belong to ${\overline{\Sigma }}$, from the discussion above it follows that $\Delta f(0,\vartheta )<0$ whenever the point with coordinate $(0,\vartheta )$ belong to $\Omega$. If we then set

$$\begin{aligned} \Omega '=\Omega \cap \left\{ (r,\vartheta ):\,|r|<-\frac{\Delta f(0,\theta )}{2C}\right\} , \end{aligned}$$

the above estimate grants us that $f(r,\theta )<f_{\textrm{max}}$ in $\Omega '\setminus {\overline{\Sigma }}$. In particular $\Omega '\cap \textrm{MAX}(f)=\Omega '\cap {\overline{\Sigma }}$. It follows that $\Omega '\cap {\overline{\Sigma }}$ is contained in the top stratum $\Sigma$, which implies in particular that $p\in \Sigma$, as desired. $\square$

Theorem 3.3 tells us that singularities of the $(n-1)$-dimensional part of the set $\textrm{MAX}(f)$ can only appear at the points where the Hessian of *f* vanish. In particular, the following corollary follows at once:

### Corollary 3.4

Let (*M*, *g*) be a real analytic Riemannian manifold, let $f:M\rightarrow {\mathbb {R}}$ be an analytic function and let $\Sigma \ne \emptyset$ be the top stratum of $\textrm{MAX}(f)$. If $\nabla ^2f$ is nowhere vanishing on ${\overline{\Sigma }}$, then

$$\begin{aligned} {\overline{\Sigma }}=\Sigma , \end{aligned}$$

thus $\Sigma$ is a complete real analytic hypersurface with empty boundary.

We emphasize that it is important to require the Hessian of *f* not to vanish on the whole closure of $\Sigma$. To illustrate this point, consider $f(x,y)=-x^2 y^2$. The function *f* is clearly analytic on the whole ${\mathbb {R}}^2$ and satisfies $|\nabla ^2f|\ne 0$ at all points of the top stratum $\Sigma =(\{x=0\}\cup \{y=0\})\setminus \{(0,0)\}$ of the set of the maxima. Nevertheless, ${\overline{\Sigma }}=\{x=0\}\cup \{y=0\}$ is not smooth, which is due to the fact that the Hessian of *f* vanishes at the singular point $(0,0)={\overline{\Sigma }}\setminus \Sigma$. Another instructive example is provided by the function $f(x,y)=-x^2 y^4$. Similar examples are easy to construct on compact manifolds *M* as well: for instance, the same behavior is shown by the function $f(x,y)=-\sin ^2 (x)\sin ^2 (y)$ on the 2-torus ${\mathbb {T}}^2=[0,\pi ]^2/\sim$.

## A Geometric Criterion for the Classification of Static Solutions

This section is devoted to the proof of Theorem 1.2. Before discussing it, let us recall briefly, following the setup carefully discussed in [12] that a static spacetime with positive cosmological constant arises as a solution of the following problem:

4.1 $$\begin{aligned} {\left\{ \begin{array}{ll} u\,\textrm{Ric}=\textrm{D}^2u+n\,u\,g, &{} \text{ in } M\\ \ \;\,\Delta u=-n\, u, &{} \text{ in } M\\ \ \ \ \ \; u>0, &{} \text{ in } M\setminus \partial M \\ \ \ \ \ \; u=0, &{} \text{ on } \partial M \end{array}\right. } \qquad \hbox {with} \ M \ \hbox {compact orientable} \ \hbox {and} \ {\mathrm R}\equiv n(n-1)\,.\nonumber \\ \end{aligned}$$

We recall from [18, 46] that a static triple $(M,g,u)$ is necessarily real analytic, so that in particular the results discussed in Section 3 apply. As a consequence, since obviously $\Delta u=-n\max _M(u)\ne 0$ on $\textrm{MAX}(u)$, we can apply Corollary 3.4 to deduce that the top stratum $\Sigma \subseteq \textrm{MAX}(u)$ is a closed real analytic hypersurface. Hence, the second fundamental form, mean curvature and induced metric are all well defined on $\Sigma$, so that the statement of Theorem 1.2 is perfectly rigorous. In the proof, we will need the following version of the Cauchy-Kovalevskaya Theorem for systems of quasilinear PDEs; we report the complete statement for the ease of reference.

### Theorem 4.1

(Cauchy-Kovalevskaya for nonlinear systems, [24]) Let $(x^1,\dots ,x^n)$ be a real analytic coordinate chart. Consider a PDE system of the form

4.2 $$\begin{aligned} {\left\{ \begin{array}{ll} \sum _{|I|=k}b_I(x,D^1 f,\dots ,D^{k-1}f)\,\partial ^I f\,+\,c(x,D^1 f,\dots ,D^{k-1}f)\,=\,0,\\ \frac{\partial ^j f}{(\partial x^1)^j}(x)\,=\,a_j(x)\ \hbox {for} x\in \{x^1=0\} \hbox {and} j=0,1,\dots ,k-1 \end{array}\right. } \end{aligned}$$

where $I=(I_1,\dots ,I_n)$ is a multiindex, $|I|=I_1+\dots +I_n$, $a_j(x)\in {\mathbb {R}}^m$, $b_I(x,D^1 f,\dots ,D^{k-1}f)\in {\mathbb {R}}^{m\times m}$, $c(x,D^1 f,\dots ,D^{k-1}f)\in {\mathbb {R}}^{m}$, $f:\Omega \rightarrow {\mathbb {R}}^m$, for some domain $\Omega \subset {\mathbb {R}}^n$ with $\Omega \cap \{x^1=0\}\ne \emptyset$. Suppose that $a_j$, $b_I$ and *c* are real analytic functions in their entries and that $b_{(k,0,\dots ,0)}$ is an invertible matrix (that is, the hypersurface $\{x^1=0\}$ is noncharacteristic). Then there is a unique real analytic solution to (4.2).

We are now ready to prove Theorem 1.2, which we rewrite here for the reader’s convenience.

### Theorem 4.2

(Geometric characterization of BK and Nariai solutions) Let (*M*, *g*, *u*) be a compact orientable *n*-dimensional, $n\geqq 3$, static triple with cosmological constant $\Lambda >0$. Suppose that the top stratum of $\textrm{MAX}(u)$ is not empty and denote by $\Sigma$ one of its connected components. Then $\Sigma$ is a compact smooth hypersurface without boundary. Furthermore, if the metric induced on $\Sigma$ by *g* is Einstein and $\Sigma$ is totally umbilic, then the universal cover of (*M*, *g*) is isometric to either a Birmingham-Kottler or a Nariai solution.

### Proof

Let us start by noticing that, given any vector field *X* on $T\Sigma$ and called $\nu$ the normal to $\Sigma$, from the static vacuum equations (4.1) we deduce

$$\begin{aligned} \textrm{Ric}(\nu ,X)\,=\,\frac{\textrm{D}^2u(\nu ,X)}{u_{\textrm{max}}}\,+\,n\,\langle \nu \,|\,X\rangle \,=\,\frac{\textrm{D}^2u(\nu ,X)}{u_{\textrm{max}}}\,=\,0, \end{aligned}$$

where the latter equality follows immediately from the general expansion (3.2). We can then apply the Gauss formula to obtain

$$\begin{aligned} 0\,=\,\textrm{Ric}(\nu ,X)\,=\,(\textrm{div}\,\textrm{h})(X)\,-\,\nabla _X\textrm{H}\end{aligned}$$

On the other hand, the umbilicity of $\Sigma$ implies $\textrm{h}=\textrm{H}/(n-1)\,g^\Sigma$, hence

$$\begin{aligned} 0\,=\,(\textrm{div}\,\textrm{h})(X)\,-\,\nabla _X\textrm{H}\,=\,\frac{1}{n-1}\nabla _X\textrm{H}\,-\,\nabla _X\textrm{H}\,=\,-\,\frac{n-2}{n-1}\,\nabla _X\textrm{H}. \end{aligned}$$

Since $n\geqq 3$, this implies that $\textrm{H}$ is constant. Furthermore, again from expansion (3.2), together with the static equations (4.1), we get

4.3 $$\begin{aligned} \textrm{Ric}(\nu ,\nu )\,=\,\frac{\textrm{D}^2u(\nu ,\nu )}{u_{\textrm{max}}}\,+\,n\,\langle \nu \,|\,\nu \rangle \,=\,-\,n\,+\,n\,=\,0. \end{aligned}$$

Substituting in the Gauss Codazzi equation,

$$\begin{aligned} {\mathrm R}^\Sigma \,&=\,{\mathrm R}\,-\,2\,\textrm{Ric}(\nu ,\nu )\,+\,\textrm{H}^2\,-\,|\textrm{h}|^2\\&=\,n(n-1)\,+\,\frac{n-2}{n-1}\,\textrm{H}^2\,. \end{aligned}$$

Let us now assume $\textrm{Ric}^\Sigma =(n-2)\lambda g^\Sigma$ for some constant $\lambda \in {\mathbb {R}}$. Then ${\mathrm R}^\Sigma \,=\,(n-1)(n-2)\,\lambda$ and we get

$$\begin{aligned} \frac{\textrm{H}}{n-1}\,=\,\sqrt{\lambda \,-\,\frac{n}{n-2}}. \end{aligned}$$

Here we are assuming that the normal $\nu$ has been chosen so that the mean curvature $\textrm{H}$ is nonnegative. As a consequence of the above formula, necessarily $\lambda \geqq n/(n-2)$ and

4.4 $$\begin{aligned} \textrm{h}\,=\,\sqrt{\lambda - \frac{n}{n-2} }\,g_\Sigma . \end{aligned}$$

Let $\Omega$ be a small open set with $\Omega \cap \Sigma \ne \emptyset$ and $\Omega \setminus \Sigma =\Omega _+\sqcup \Omega _-$, with the normal $\nu$ pointing towards $\Omega _+$. We have already mentioned in Theorem 3.1 that the signed distance function

$$\begin{aligned} r(x)\,=\, {\left\{ \begin{array}{ll} +d(x,\Sigma ), &{} \hbox { if } x\in {\overline{\Omega }}_+,\\ -d(x,\Sigma ), &{} \hbox { if } x\in {\overline{\Omega }}_-, \end{array}\right. } \end{aligned}$$

is an analytic function in $\Omega$. Since clearly $|\nabla r|\equiv 1$ in $\Omega$, with respect to coordinates $(r,\vartheta )=(r,\vartheta ^1,\dots ,\vartheta ^{n-1})$, with $\vartheta ^1,\dots ,\vartheta ^{n-1}$ coordinates on the hypersurface $\Sigma \cap \Omega$, we have

$$\begin{aligned} g\,=\,dr\otimes dr\,+\,g_{ij}\,d\vartheta ^i\otimes d\vartheta ^j, \end{aligned}$$

where the coefficients $g_{ij}$ are functions of the coordinates $(r,\vartheta )$. Since we want to invoke the Cauchy-Kovalevskaya Theorem as stated in Theorem 4.1, it is convenient to restrict our attention to a subdomain of $\Omega$ that can be identified with a cylinder $(-\varepsilon ,\varepsilon )\times {\mathbb {B}}^{n-1}$. With a slight abuse of notation, we think of the coordinates $(r,\vartheta )$ as varying in $(-\varepsilon ,\varepsilon )\times {\mathbb {B}}^{n-1}$ and as a consequence, all quantities are considered as pulled back on the same domain.

With respect to the coordinates $(r,\vartheta )$, observing that the normal $\nu$ coincides with $\partial /\partial r$ on $\Sigma$, we easily see that the second fundamental form of a level set of *r* satisfies

$$\begin{aligned} \textrm{h}_{ij}\,=\,\textrm{D}^2_{ij} r\,=\,-\Gamma _{ij}^r\,=\,\frac{1}{2}\,\partial _r g_{ij}. \end{aligned}$$

Formula (4.4) tells us that4.5We have also the following initial conditions:4.6We would like to apply the Cauchy-Kovalevskaya Theorem 4.1 to the function

$$\begin{aligned} f\,:\,(-\varepsilon ,\varepsilon )\times {\mathbb {B}}^{n-1}\,&\longrightarrow \,{\mathbb {R}}^{1+\frac{n(n-1)}{2}}\\ x=(r,\vartheta )\,&\longmapsto \,\Big (u(x)\,,\,g_{ij}(x)\Big )\, \end{aligned}$$

by showing that *f* satisfies a PDE as in (4.2) coming from the equations (4.1) and the initial conditions (4.5), (4.6) on $\Sigma =\{r=0\}$. To this end, let us rewrite problem (4.1) more explicitly in terms of the derivatives of *u* and the metric $g$. We recall that, in any coordinate system, the components of the Ricci tensor satisfy

$$\begin{aligned} {\mathrm R}_{\alpha \beta }\, =\,-\frac{g^{\mu \eta }}{2}\left[ \partial _{\mu \eta }^2 g_{\alpha \beta }\, +\,\partial _{\alpha \beta }^2 g_{\mu \eta }\,-\,\partial _{\mu \alpha }^2 g_{\eta \beta }\,-\,\partial _{\eta \beta }^2 g_{\mu \alpha }\right] \,+\,\hbox { lower order terms}, \end{aligned}$$

where the lower order terms are polynomial functions of the components of *g*, the inverse of *g* and their first derivatives. In particular, with respect to the coordinates $(r,\vartheta ^1,\dots ,\vartheta ^{n-1})$, the components ${\mathrm R}_{ij}$ of the Ricci tensor satisfy

$$\begin{aligned} {\mathrm R}_{ij}\,&=\,-\frac{g^{\mu \eta }}{2}\left[ \partial _{\mu \eta }^2 g_{ij}\,+\,\partial _{ij}^2 g_{\mu \eta }\,-\,\partial _{\mu i}^2 g_{\eta j}\,-\,\partial _{\eta j}^2 g_{\mu i}\right] \,+\,\hbox { lower order terms}\\&=\,-\frac{1}{2}\,\partial ^2_{rr}g_{ij}-\frac{g^{ab}}{2}\left[ \partial _{ab}^2 g_{ij}\,+\,\partial _{ij}^2 g_{ab}\,-\,\partial _{a i}^2 g_{b j}\,-\,\partial _{b j}^2 g_{a i}\right] \,+\,\hbox { lower order terms} \end{aligned}$$

where $\mu ,\eta$ take the values $r,1,\dots ,n-1$ whereas *a*, *b* take only the values $1,\dots ,n-1$. Again the lower order terms are polynomial functions of the components of $g$, the inverse of $g$ and their first derivatives. Notice that, since $g$ is nonsingular, the inverse of $g$ is well defined everywhere and its components $g^{ij}$ are analytic functions of the components $g_{ij}$. Therefore, the lower order terms appearing above are analytic functions of $g$ and the first derivatives of $g$. We can now rewrite problem (4.1) as

4.7 $$\begin{aligned} {\left\{ \begin{array}{ll} -\frac{u}{2}\,\partial ^2_{rr}g_{ij}-\frac{u}{2}g^{ab}\left[ \partial _{ab}^2 g_{ij}\,+\,\partial _{ij}^2 g_{ab}\,-\,\partial _{a i}^2 g_{b j}\,-\,\partial _{b j}^2 g_{a i}\right] \,-\partial ^2_{ij}u\,=\,\hbox {lower order terms},\\ \partial ^2_{rr}u\,+\,g^{ab}\,\partial ^2_{ab}u\,=\,\hbox {lower order terms}, \end{array}\right. } \end{aligned}$$

where the lower order terms appearing in (4.7) are analytic functions depending on *u*, $g$ and their first derivatives. In order to apply the Cauchy-Kovalevskaya Theorem 4.1 to problem (4.7) with initial conditions (4.5), (4.6), it only remains to show that the surface $\Sigma$ is noncharacteristic for the system (4.7). In other words, we have to check that the matrix

4.8 $$\begin{aligned} b_{(2,0,\dots ,0)}\,=\, \begin{bmatrix} 0 &{} -u/2 &{} 0 &{} \cdots &{} 0 \\ 0 &{} 0 &{} -u/2 &{} \cdots &{} 0 \\ \vdots &{} \vdots &{} \vdots &{} \ddots &{} \vdots \\ 0 &{} 0 &{} 0 &{} \cdots &{} -u/2 \\ 1 &{} 0 &{} 0 &{} \cdots &{} 0 \end{bmatrix} \end{aligned}$$

is invertible. Since $u=\max _M(u)> 0$ on $\Sigma$, this is trivially true, hence the Cauchy-Kovalevskaya Theorem can be applied, telling us that there is a unique analytic solution to (4.7). On the other hand, the BK and Nariai solutions also solve (4.7). Moreover, it can be checked (see (5.19) and (5.13) below) that, for any $\lambda >n/(n-2)$ there is a value $0<m<m_{\textrm{max}}$ such that the BK solution with mass *m* satisfies the initial conditions (4.5), (4.6) with respect to the chosen $\lambda$, whereas the Nariai solution satisfies the initial conditions (4.5), (4.6) with respect to $\lambda =n/(n-2)$ (more explicitly, on the models $\lambda$ and *m* are related by $\lambda =[(n-2)m]^{-2/n}$). It follows that our solution has to coincide with a model solution inside $\Omega$. This argument shows that a tubular neighborhood of $\Sigma$ is locally isometric to a tubular neighborhood of the set of the maxima of a model solution. In other words, there exists $0<a<u_{\textrm{max}}$ and a Riemannian covering of $(M,g,u)\cap \{u>a\}$ by a BK or Nariai solution. If $a\ne 0$, then the matrix (4.8) is invertible on the hypersurface $\{u=a\}$, so we could apply the Cauchy-Kovalevskaya Theorem again on $\{u=a\}$. It follows that the Riemannian covering can be extended up to the points where $u=0$, that is, up to the horizons. Therefore, the whole $(M,g,u)$ is isometric to a quotient of a BK or Nariai solution. $\square$

## Analysis of the Interfaces and Black Hole Uniqueness Theorem

This section is mainly devoted to the proof of Theorem 5.1 below, which will be a crucial ingredient, together with Theorem 4.2, in the proof of Theorem 1.1.

We start with a quick reminder of some definitions that will be helpful in the following discussion. For more details, we refer the reader to [12]. A connected component *N* of $M\setminus \textrm{MAX}(u)$ will be called *region*. A component *S* of $\partial M$ that belongs to the region *N* is called *horizon* of *N* and its *surface gravity* is the constant value of $|\nabla u|/\max _M(u)$ on *S*. We will distinguish different types of regions depending on the value of the surface gravity at the horizons of *N*. Namely, a region will be called *outer*, *inner* or *cylindrical*, depending on whether the maximum surface gravity of the horizons of *N* is smaller than, greater than or equal to $\sqrt{n}$, respectively. Given a region *N* of a solution $(M,g,u)$ of (4.1), the *virtual mass* $\mu (N,g,u)$ is defined as the mass of the model solution that would be responsible for the maximum of the surface gravities detected at the horizons of *N*, see [12, Definition 3]. The virtual mass is always a number between 0 and $m_{\textrm{max}}$, defined as

5.1 $$\begin{aligned} m_{\textrm{max}}=\sqrt{\frac{(n-2)^{n-2}}{n^n}}. \end{aligned}$$

**Fig. 1:**
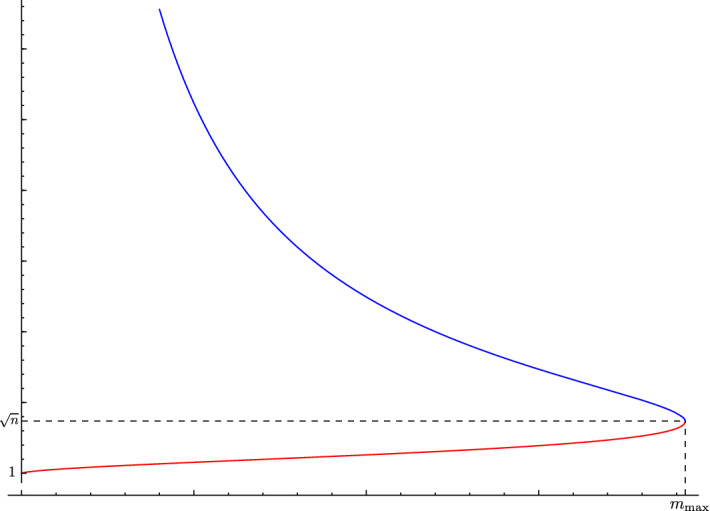
Plot of the surface gravities $|\nabla u|/\max _M(u)$ of the two boundaries of the Schwarzschild–de Sitter solution as functions of the mass *m*. The red line represents the surface gravity of the cosmological horizon $\partial M_+ = \{r = r_+(m)\}$, whereas the blue line represents the surface gravity of the black hole horizon $\partial M_- = \{r = r_-(m)\}$. Notice that for $m = 0$ we recover the constant value $|\nabla u| = 1$ of the surface gravity on the cosmological horizon of the de Sitter solution. The other special situation is when $m = m_{\textrm{max}}$. In this case the plot assigns to $m_{\textrm{max}}$ the unique value $\sqrt{n}$ achieved by the surface gravity on both the horizons of the Nariai solution

The meaning of these definitions and the interplay between virtual mass and surface gravity on the models can be appreciated in Fig. 1.

It has been proven in [11, Theorem 2.3] that the virtual mass is always well defined and it is equal to zero only on the de Sitter spacetime. Finally, given two different regions *A* and *B*, if ${\overline{A}}\cap {\overline{B}}=:\Sigma \ne \emptyset$ then it follows from Corollary 3.4 that $\Sigma$ is a real analytic hypersurface with empty boundary. The hypersurface $\Sigma$ will be called *interface*.

We are now ready to state the following result:

### Theorem 5.1

(Analysis of the interfaces) Let $(M,g,u)$ be a solution to problem (4.1). Let *A*, *B* be two connected components of $M\setminus \textrm{MAX}(u)$ such that ${\overline{A}}\cap {\overline{B}}=:\Sigma \ne \emptyset$.

(i) If *A* is outer, then *B* is necessarily inner and
  $$\begin{aligned} \mu (A,g,u)\,\geqq \,\mu (B,g,u). \end{aligned}$$
  Moreover, if $\mu (A,g,u)=\mu (B,g,u)=m$ then $\Sigma$ is an umbilical CMC hypersurface and the metric induced by *g* on $\Sigma$ has constant scalar curvature.
(ii) If *A* is cylindrical, then *B* is inner or cylindrical and obviously
  $$\begin{aligned} m_{\textrm{max}}\,=\,\mu (A,g,u)\,\geqq \,\mu (B,g,u). \end{aligned}$$
  Moreover, if $\mu (A,g,u)=\mu (B,g,u)=m_{\textrm{max}}$ (that is, *B* is cylindrical) then $\Sigma$ is a totally geodesic hypersurface and the metric induced by *g* on $\Sigma$ has constant scalar curvature.

Theorem 5.1 can be seen as a higher dimensional version of [12, Theorem 7.2 and Theorem 8.15], although the strategy of the proof proposed here is new. In the proof, we will actually compute explicitly the value of mean curvature, second fundamental form and scalar curvature of $\Sigma$ in the rigidity case (see (5.18),  (5.19) and (5.20) for case (*i*) and (5.13),  (5.14) for case (*ii*)). Once Theorem 5.1 is established, Theorem 1.1 follows at once (see Subsection 5.4), thanks to the geometric criterion established in Theorem 4.2.

### Analytic preliminaries

One of the fundamental ingredients in the proof of Theorem 5.1 is the gradient estimate for the lapse function proven in [12, Proposition 3.3]. In Proposition 5.2 we recall that result and we outline the proof, as it represents an important application of the Reverse Łojasiewicz inequality, and more precisely of Corollary 2.3. Our aim is to show that the gradient of the lapse function $|\nabla u|_g$ is pointwise controlled by the corresponding quantity $|\nabla u_m|_{g_m}$ on the model solution of the same mass *m*. Here we have denoted by $u_m$ and $g_m$ the lapse function and metric of the BK solution (1.1) with mass $m\in (0,m_{\textrm{max}})$, which we recall here for the reader’s convenience:

$$\begin{aligned} g_m = \frac{dr^2}{u_m^2} + r^2 g_\Sigma , \quad u_m = \sqrt{1 -r^2 - \frac{2m}{r^{n-2}}} . \end{aligned}$$

In order to write a clean statement, we first need to introduce some more notation. Given $m\in (0,m_{\textrm{max}})$, we denote by

$$\begin{aligned} u_{\textrm{max}}(m)=\sqrt{1-\left( \frac{m}{m_{\textrm{max}}}\right) ^{2/n}} \end{aligned}$$

the maximum value of the lapse function $u_m$ of the Schwarzschild–de Sitter solution with mass *m*. We then use the Implicit Function Theorem to show that, for any value $m\in (0,m_{\textrm{max}})$ and for any region *N*, once *u* is normalized so that $\max _M(u)=u_{\textrm{max}}(m)$, there exists a so called *pseudo-radial function* $\Psi : N \longrightarrow {\mathbb {R}}$, satisfying the relationships

5.2 $$\begin{aligned} u(p)&= \sqrt{1-\Psi ^2(p)- 2m\Psi ^{2-n}(p)} \quad \hbox {for every} p \in N\nonumber \\ \Psi \equiv r_{\pm }(m)&\quad \hbox {on} N\cap \partial M \qquad \hbox {and} \qquad \Psi \equiv r_0(m) \quad \hbox {on} {\overline{N}}\cap \textrm{MAX}(u) \, . \end{aligned}$$

Here $r_-(m)<r_+(m)$ are defined as the radii of the two horizons of the BK solutions of mass *m*, whereas $r_0(m)=[(n-2)m]^{1/n}$ represents the radius of $\textrm{MAX}(u)$ in the model solution, and the sign $+$ or − in the boundary condition $\Psi \equiv r_{\pm }(m)$ depends on whether *N* is an outer or inner region, respectively. The definition of $\Psi$ has been chosen in order to mimic the behaviour of the radial coordinate on the model solutions. Further discussions on its interpretation and well posedness can be found in [12, Subsection 2.1]. The pseudo-radial function is then exploited to prove the following fundamental gradient estimate:

#### Proposition 5.2

(Gradient Estimate) Let $(M,g,u)$ be a solution of (4.1), and let *N* be an outer or inner region with virtual mass $m=\mu (N,g,u)<m_{\textrm{max}}$. Normalize *u* so that $\max _M(u)=u_{\textrm{max}}(m)$. Then it inequality holds that

5.3 $$\begin{aligned} |\nabla u|_g \,\, \leqq \,\, |\nabla u_m|_{g_m} \circ \Psi , \end{aligned}$$

where the function $|\nabla u_m|_{g_m}$ on the right hand side has to be understood as the function of one real variable that associates to some $t \in [r_-(m),r_+(m)]$ the constant value assumed by the length of the gradient of $u_m$ on the set $\{ |x| = t \}$.

#### Proof

The full proof of this fact has been given, with a slightly different formalism, in [12, Proposition 3.3]. Here we recall the main points. The argument leading to (5.3) is quite delicate and relies on the fact that the quantity

5.4 $$\begin{aligned} W \, = \, \frac{\Psi }{|\nabla u_m|_{g_m} \circ \Psi } \left( |\nabla u_m|^2_{g_m} \circ \Psi - |\nabla u|^2 \right) \end{aligned}$$

satisfies a convenient elliptic partial differential inequality on *N*, namely

5.5 $$\begin{aligned} \begin{aligned}&\Delta _{\left( {g}/{\Psi ^2}\right) } W - \frac{(n-2)u^2 \Psi ^{n-2} + \Psi ^n - (n-2)m}{u \,[\Psi ^n-(n-2)m]} \,\big \langle \textrm{d}u \, \big | \textrm{d}W \big \rangle _{\!(g/\Psi ^2)}\\&\quad - n(n-2)m \frac{u^2 \Psi ^n }{[\Psi ^n-(n-2)m]^2} \left[ \frac{ (n-2) |\nabla u_m|^2_{g_m} \circ \Psi + (n+2) |\nabla u|^2 }{ |\nabla u_m|^2_{g_m} \circ \Psi } \right] W \, \leqq \, 0 \,, \end{aligned} \end{aligned}$$

where $\Delta _{\left( {g}/{\Psi ^2}\right) }$ and $\langle \cdot | \cdot \rangle _{\left( {g}/{\Psi ^2}\right) }$ represent the Laplacian and the scalar product of the conformally related metric $\Psi ^{-2}g$. The proof of inequality (5.5) was presented in [12, Formula (3.24)] with a different formalism. The equivalence between (5.5) and [12, Formula (3.24)] is shown in the appendix.

One observes that $W \geqq 0$ on $N\cap \partial M$ by construction, since we are comparing with a model solution $(M,g_m,u_m)$ having the same mass as (*N*, *g*, *u*) (see [12, Lemma 2.2] for details). We now want to show that the limit

$$\begin{aligned} \lim _{p\rightarrow \textrm{MAX}(u)}W(p)\,=\,\lim _{p\rightarrow \textrm{MAX}(u)} \Psi \,\left( |\nabla u_m|_{g_m} \circ \Psi -\frac{|\nabla u|^2 }{|\nabla u_m|_{g_m} \circ \Psi } \right) \end{aligned}$$

is equal to zero. To this end, we first notice that one has an explicit formula for the gradient of the lapse function of the model solution, namely

$$\begin{aligned} |\nabla u_m|_{g_m} \circ \Psi \,=\,\Psi \,\left| 1-\left( \frac{r_0(m)}{\Psi }\right) ^{\!n}\right| . \end{aligned}$$

Recalling the definition of $\Psi$, it is easily seen that $|\nabla u_m|_{g_m}\circ \Psi$ goes to zero at the same rate of $\sqrt{\max _M(u)-u}$ as we approach $\textrm{MAX}(u)$. Therefore, there exists a constant $C>0$ such that

$$\begin{aligned} \lim _{p\rightarrow \textrm{MAX}(u)}W(p)\,=\,-\,C\lim _{p\rightarrow \textrm{MAX}(u)} \frac{|\nabla u|^2 }{\sqrt{u_{\textrm{max}}-u}}. \end{aligned}$$

It follows then from the Reverse Łojasiewicz Inequality (more precisely from Corollary 2.3) that $W(p) \rightarrow 0$, as $p \rightarrow \textrm{MAX} (u)$. In particular, applying the Minimum Principle on the region $\Omega ^\varepsilon = \{|W| \geqq \varepsilon \} \cap N$, for every sufficiently small $\varepsilon >0$, one deduces that $\min _{\Omega ^\varepsilon } \!W \!\geqq - \varepsilon$, and in turn the desired gradient estimate. $\square$

We will also need some estimate for the lapse function and the pseudo-radial function near the interface of two regions. We start by recalling the following expansion, already proven in [12, Proposition 2.6], together with a short proof for completeness.

#### Proposition 5.3

Let $(M,g,u)$ be a solution to problem (4.1) and let *A*, *B* be two connected components of $M\setminus \textrm{MAX}(u)$ with ${\overline{A}}\cap {\overline{B}}=:\Sigma \ne \emptyset$. Then, the signed distance

5.6 $$\begin{aligned} r(x)\,=\, {\left\{ \begin{array}{ll} + \, d(x,\Sigma ), &{} \text { if } x\in {\overline{A}},\\ - \, d(x,\Sigma ), &{} \text { if } x\in {\overline{B}}\, \end{array}\right. } \end{aligned}$$

is an analytic function in a neighborhood of $\Sigma$ and the function *u* admits the expansion

5.7 $$\begin{aligned} u\,=\,\max _M(u)\,\left[ 1-\frac{n}{2}\,r^2\,+\,\frac{n}{6}\,\textrm{H}\,r^3 \,-\,\frac{n}{24}\left( 2\,|\mathring{\textrm{h}}|^2\,+\,\frac{n+1}{n-1}\,\textrm{H}^2\,-\,n\right) r^4\,+{\mathcal {O}}(r^5)\right] ,\nonumber \\ \end{aligned}$$

where $\textrm{H},\mathring{\textrm{h}}$ are the mean curvature and traceless second fundamental form of $\Sigma$ with respect to the normal $\nu$ pointing towards *A*.

#### Proof

From Theorem 3.1 we get the analyticity of the signed distance function *r* and the following expansion around $\Sigma$:

$$\begin{aligned} u\,=\,\max _M(u)\,\left[ 1-\frac{n}{2}\,r^2\,+\,\frac{n}{6}\,\textrm{H}\,r^3 \,-\,\frac{n}{24}\left( |\textrm{h}|^2\,+\,2\,\textrm{H}^2\,+\,{\mathrm R}\,-\,{\mathrm R}^\Sigma \,-\,n\right) r^4\,+{\mathcal {O}}(r^5)\right] . \end{aligned}$$

We also know from (4.3) that $\textrm{Ric}(\nu ,\nu )=0$ on $\Sigma$. We can then apply the Gauss-Codazzi equation to obtain

$$\begin{aligned} |\textrm{h}|^2\,+\,2\,\textrm{H}^2\,+\,{\mathrm R}\,-\,{\mathrm R}^\Sigma \,&=\,2\,\textrm{Ric}(\nu ,\nu )\,+\,2\,|\textrm{h}|^2\,+\,\textrm{H}^2\\&=\,2\,|\mathring{\textrm{h}}|^2\,+\,\frac{2}{n-1}\textrm{H}^2\,+\,\textrm{H}^2\\&=\,2\,|\mathring{\textrm{h}}|^2\,+\,\frac{n+1}{n-1}\,\textrm{H}^2\,. \end{aligned}$$

Substituting in the expansion for *u* above, we obtain (5.7). $\square$

To put our technique in a perspective, it is worth recalling that Propositions 5.2 and 5.3 were already present in [12] and have been reported for the reader’s convenience. What follows from now on represents instead an original contribution of the present paper. Here, our strategy diverges from the one in [12], as the argument in [12] was based on a delicate integration by parts along the interface, whereas the following results and propositions are essentially based on the power series expansion of some relevant quantities in a neighborhood of $\textrm{MAX}(u)$. The first step is indeed a detailed expansion of the pseudo-radial function about the interface between two different regions.

#### Proposition 5.4

Let $(M,g,u)$ be a solution to problem (4.1), let *A*, *B* be two connected components of $M\setminus \textrm{MAX}(u)$ such that ${\overline{A}}\cap {\overline{B}}=:\Sigma \ne \emptyset$ and let *r* be the signed distance to $\Sigma$ defined as in (5.6). Fix $m\in (0,m_{\textrm{max}})$ and normalize *u* so that $\max _M(u)=u_{\textrm{max}}(m)$. Let $\Psi :{\overline{A}}\cup {\overline{B}}\rightarrow {\mathbb {R}}$ be the pseudo-radial function defined by (5.2) with respect to a parameter $m\in (0,m_{\textrm{max}})$ and with boundary conditions $\Psi =r_+(m)$ on $A\cap \partial M$, $\Psi =r_-(m)$ on $B\cap \partial M$, $\Psi =r_0(m)$ on $\Sigma$. Then the function $\Psi$ is ${\mathscr {C}}^3$ in a neighborhood of $\Sigma$ and the following expansion holds:

5.8 $$\begin{aligned}{} & {} \Psi \,=\,u_{\textrm{max}}(m)\bigg [\frac{r_0(m)}{u_{\textrm{max}}(m)}\,+\,r\,+\,\frac{n-1}{6}\,K\,r^2\,+\,\nonumber \\{} & {} \,+\,\frac{1}{12}\left( |\mathring{\textrm{h}}|^2\,-\,2\,n\,+\,\frac{n-1}{9}\,K\,\left( (n-4)\frac{u_{\textrm{max}}(m)}{r_0(m)}-(n+2)\frac{\textrm{H}}{n-1}\right) \right) r^3\,+\,o(r^3)\bigg ].\nonumber \\ \end{aligned}$$

Here

$$\begin{aligned} K=\frac{u_{\textrm{max}}(m)}{r_0(m)}\,-\,\frac{\textrm{H}}{n-1}, \end{aligned}$$

and $\textrm{H}$ is the mean curvature of $\Sigma$ with respect to the normal $\partial /\partial r$.

#### Proof

In order to simplify notations, throughout this proof we will avoid to make the dependence on *m* explicit. Namely, we will write $u_{\textrm{max}}$ and $r_0$ instead of $u_{\textrm{max}}(m)$ and $r_0(m)$.

We first observe that the boundary conditions imposed on $\Psi$ have been chosen in such a way that we can invoke [12, Proposition 2.7], which tells us that $\Psi$ is ${\mathscr {C}}^3$ in a neighborhood of $\Sigma$. Therefore, it only remains to compute the explicit expansion of $\Psi$. We start by recalling that $\Psi =r_0$ on $\textrm{MAX}(u)$, and then we write

5.9 $$\begin{aligned} \Psi \,=\,r_0\,+\,v\,r\,+\,w\,r^2\,+\,z\,r^3\,+\,F, \end{aligned}$$

where *v*, *w*, *z* are functions of the coordinates $x^2,\dots ,x^n$ only, and $F=o(r^3)$. Now we compute the expansions of the left and right hand sides of the relation $u^2\,=\,1-\Psi ^2-2m\Psi ^{2-n}$ to obtain information on the functions *v*, *w*, *z*. Taking the square of (5.7), we get

5.10 $$\begin{aligned} u^2\,=\,u_{\textrm{max}}^2\,\left[ 1-n\,r^2\,+\,\frac{n}{3}\,\textrm{H}\,r^3 \,+\,\frac{n}{12}\left( 4\,n\,-\,2\,|\mathring{\textrm{h}}|^2\,-\,\frac{n+1}{n-1}\,\textrm{H}^2\right) r^4\,+o(r^4)\right] .\nonumber \\ \end{aligned}$$

On the other hand, with some lengthy (but standard) computations, from (5.9) one obtains

$$\begin{aligned}{} & {} \Psi ^2\,=\,r_0^2\bigg [1\,+\,2\,\frac{v}{r_0}\,r\,+\,\left( 2\,\frac{w}{r_0}\,+\,\frac{v^2}{r_0}\right) r^2\,+\,2\,\left( \frac{z}{r_0}\,+\,\frac{v\,w}{r_0^2}\right) r^3\\{} & {} \quad +\,\left( 2\,\frac{v\,z}{r_0^2}\,+\,\frac{w^2}{r_0^2}\right) r^4\,+\,2\,\frac{F}{r_0}\,+\,o(r^4)\bigg ],\\{} & {} \Psi ^{2-n}\,=\,\frac{r_0^2}{m}\bigg [\frac{1}{n-2}-\frac{v}{r_0}\,r\,+\,\left( \frac{n-1}{2}\,\frac{v^2}{r_0^2}\,-\,\frac{w}{r_0}\right) r^2\,\\{} & {} \quad +\,\left( -\frac{n(n-1)}{6}\,\frac{v^3}{r_0^3}\,+\,(n-1)\frac{v\,w}{r_0^2}\,-\,\frac{z}{r_0}\right) r^3\\{} & {} \quad +(n-1)\left( \frac{n(n+1)}{24}\frac{v^4}{r_0^4}\,-\,\frac{n}{2}\,\frac{v^2\,w}{r_0^3}\,+\,\frac{v\,z}{r_0^2}\,+\,\frac{1}{2}\,\frac{w^2}{r_0^2}\right) r^4\,-\,\frac{F}{r_0}\,+\,o(r^4)\,\bigg ]. \end{aligned}$$

From these expansions we get

$$\begin{aligned}{} & {} 1-\Psi ^2-2m\Psi ^{2-n}\,=\,u_{\textrm{max}}^2\,-\,n\,v^2\,r^2\,+\,n\,\left( \frac{n-1}{3}\,\frac{v^3}{r_0}\,-\,2\,v\,w\right) r^3\\{} & {} \quad +\,n\left( -\frac{(n-1)(n+1)}{12}\frac{v^4}{r_0^2}\,+\,(n-1)\frac{v^2\,w}{r_0}\,-\,2\,v\,z\,-\,w^2\right) r^4\,+\,o(r^4). \end{aligned}$$

Comparing with (5.10), we obtain

$$\begin{aligned}&v^2\,=\,u_{\textrm{max}}^2\,,\\&\frac{n-1}{3}\,\frac{v^3}{r_0}\,-\,2\,v\,w\,=\,\frac{\textrm{H}}{3}\,u_{\textrm{max}}^2\,,\\&-\frac{(n-1)(n+1)}{12}\frac{v^4}{r_0^2}\,+\,(n-1)\frac{v^2\,w}{r_0}\,-\,2\,v\,z\,-\,w^2\,\\ {}&\quad =\,\frac{u_{\textrm{max}}^2}{12}\left( 4\,n\,-\,2\,|\mathring{\textrm{h}}|^2\,-\,\frac{n+1}{n-1}\,\textrm{H}^2\right) \,. \end{aligned}$$

From the first identity we get $v=\pm u_{\textrm{max}}$. To decide the sign, we recall the definitions of $\Psi$ and *r* and we notice that they have been chosen in such a way that $\Psi <r_0$ when $r>0$ and $\Psi >r_0$ when $r<0$. Therefore, recalling (5.9), the correct choice is to take a positive *v*, hence $v=u_{\textrm{max}}$. Substituting in the second and third identity, we easily compute the corresponding expressions for *w* and *z* and we recover formula (5.8), as wished. $\square$

### Proof of Theorem 5.1–(*ii*)

Let us start the proof of Theorem 5.1 by first addressing the cylindrical case, since it presents far less technical difficulties while containing all the main ideas. Given a solution $(M,g,u)$ of problem (4.1), according to [12], we normalize *u* so that $\max _M(u)=1$. For a cylindrical region $N\subseteq M\setminus \textrm{MAX}(u)$, we have a gradient estimate in the same spirit of (5.3). In fact, [12, Proposition 8.2] tells us that $|\nabla u|$ is bounded by the norm of the gradient of the lapse function of the Nariai solution on the corresponding level set. More explicitly, we have the following inequality:

5.11 $$\begin{aligned} \frac{|\nabla u|^2}{n(1-u^2)}\,\leqq \,1. \end{aligned}$$

This inequality can then be employed to prove

#### Proposition 5.5

Let $(M,g,u)$ be a solution to problem (4.1). Suppose that there are two regions *A*, *B* such that $\Sigma :={\overline{A}}\cap {\overline{B}}$ is not empty. Let $\textrm{H}$ be the mean curvature of $\Sigma$ with respect to the normal pointing inside *A*. If *A* is a cylindrical region, then

$$\begin{aligned} \textrm{H}\,\geqq \,0. \end{aligned}$$

#### Proof

Since we have normalized *u* so that its maximum is 1, from (5.7) we obtain the following expansions in terms of the signed distance *r*:

$$\begin{aligned} |\nabla u|^2\,&=\,n^2\,r^2\,\left[ 1\,-\,\textrm{H}\,r\,+\,{\mathcal {O}}(r^2)\right] \,,\\ u\,&=\,\left[ 1-\frac{n}{2}\,r^2\,+\,\frac{n}{6}\,\textrm{H}\,r^3\,+{\mathcal {O}}(r^4)\right] \,. \end{aligned}$$

Here $\textrm{H}$ is the mean curvature of $\Sigma$ with respect to the unit normal $\nu =\partial /\partial r$ (which is the one pointing inside *A*). An easy computation now gives

5.12 $$\begin{aligned} \frac{|\nabla u|^2}{n(1-u^2)}\,=\,1\,-\,\frac{2}{3}\,\textrm{H}\,r\,+\,{\mathcal {O}}(r^2). \end{aligned}$$

By definition, *r* is positive in *A* and negative in *B*, hence, in order for (5.11) to hold, it must be $\textrm{H}\geqq 0$, as wished. $\square$

Now we proceed to the proof of Theorem 5.1 in the cylindrical case. Since *A* is cylindrical, from Proposition 5.5 we get $\textrm{H}\geqq 0$, where $\textrm{H}$ is the mean curvature of $\Sigma$ with respect to the normal pointing inside *A*. The fact that *B* cannot be outer follows now from Proposition 5.6 (proven in the next subsection) that tells us that if *B* were outer then the mean curvature of $\Sigma$ with respect to the opposite normal (the one pointing inside *B*) would be positive, which is a contradiction. If *B* is cylindrical, applying again Proposition 5.5 to both *A* and *B*, we get

$$\begin{aligned} 0\,\leqq \,\textrm{H}\,\leqq \, 0. \end{aligned}$$

Therefore, $\textrm{H}=0$, as wished. Substituting this information inside the expansions for *u* and $|\nabla u|^2$, we can refine (5.12) and compute that, if $\textrm{H}=0$ on $\Sigma$, then

$$\begin{aligned} \frac{|\nabla u|^2}{n(1-u^2)}\,=\,1\,+\,\frac{1}{2}|\mathring{\textrm{h}}|^2\,r^2\,+\,o(r^2). \end{aligned}$$

From (5.11) it follows then that $|\mathring{\textrm{h}}|=0$, that is,

5.13 $$\begin{aligned} \textrm{h}=\frac{\textrm{H}}{n-1}\,g^\Sigma \,=\,0. \end{aligned}$$

We also know from (5.3) that $\textrm{Ric}(\nu ,\nu )=0$ on $\Sigma$. Substituting these pieces of information in the Gauss-Codazzi equation, we get

5.14 $$\begin{aligned} {\mathrm R}^\Sigma \,=\,{\mathrm R}\,-\,2\,\textrm{Ric}(\nu ,\nu )\,+\,\textrm{H}^2\,-\,|\textrm{h}|^2\,=\,n(n-1). \end{aligned}$$

This concludes the proof.

### Proof of Theorem 5.1–(*i*)

We now focus on the noncylindrical case. Let $(M,g,u)$ be a solution to problem (4.1) and consider a region with virtual mass

$$\begin{aligned} m\,=\,\mu (N,g,u)\,<\,m_{\textrm{max}}. \end{aligned}$$

We start by rewriting more explicitly the gradient estimate (5.3), by writing the value of $|\nabla u_m|_{g_m} \circ \Psi$ as a function of the pseudo-radial function

$$\begin{aligned} |\nabla u_m|_{g_m} \circ \Psi \,=\,\Psi \,\big | 1-(n-2)m\Psi ^{-n} \big |. \end{aligned}$$

In particular, we can rewrite (5.3) as

5.15 $$\begin{aligned} \frac{|\nabla u|^2}{\Psi ^2\left[ 1-(n-2)m\Psi ^{-n}\right] ^2}\,\leqq \,1. \end{aligned}$$

The aim of this subsection is to compare (5.15) with the expansions discussed in Subsection 5.1 in order to deduce some consequences on the geometry of the interface $\Sigma$. More specifically, we now prove the following analogue of Proposition 5.5:

#### Proposition 5.6

Let $(M,g,u)$ be a solution to problem (4.1). Suppose that there are two regions *A*, *B* such that $\Sigma :={\overline{A}}\cap {\overline{B}}$ is not empty, and let $m_A,m_B$ be the virtual masses of *A*, *B*. Let $\textrm{H}$ be the mean curvature of $\Sigma$ with respect to the normal pointing inside *A*.

- If *A* is an outer region, then
  $$\begin{aligned} \frac{\textrm{H}}{n-1}\,\geqq \,\frac{u_{\textrm{max}}(m_A)}{r_0(m_A)}. \end{aligned}$$
- If *B* is an inner region, then
  $$\begin{aligned} \frac{\textrm{H}}{n-1}\,\leqq \,\frac{u_{\textrm{max}}(m_B)}{r_0(m_B)}. \end{aligned}$$

#### Proof

Let *r* be the signed distance function defined in (5.6). We first observe that the metric $g$ can be written in terms of coordinates $(r,\vartheta ^1,\dots ,\vartheta ^{n-1})$ as

$$\begin{aligned} g\,=\,dr\otimes dr\,+\,{g{}^{\!\top }}_{ij}\,d\vartheta ^i\otimes d\vartheta ^j. \end{aligned}$$

Starting from (5.7), one easily computes the following expansion for $|\nabla u|^2$ along $\Sigma$ as

$$\begin{aligned} |\nabla u|^2\,&=\,\left( \frac{\partial u}{\partial r}\right) ^2\,+\,({g{}^{\!\top }})^{ij}\frac{\partial u}{\partial \vartheta ^i}\,\frac{\partial u}{\partial \vartheta ^j}\\&=\,n^2\,u_{\textrm{max}}^2(m)\,r^2\,\left[ 1\,-\,\textrm{H}\,r\,+\,{\mathcal {O}}(r^2)\right] \,. \end{aligned}$$

where $\textrm{H}$ is the mean curvature of $\Sigma$ with respect to the unit normal $\nu =\partial /\partial r$ (which is the one pointing inside *A*). We also recall from (5.8) the expansion

$$\begin{aligned} \Psi \,&=\,r_0(m)\,\left[ 1\,+\,\frac{u_{\textrm{max}}(m)}{r_0(m)}\,r\,+\,\frac{n-1}{6}\,\frac{u_{\textrm{max}}(m)}{r_0(m)}\,\left( \frac{u_{\textrm{max}}(m)}{r_0(m)}-\frac{\textrm{H}}{n-1}\right) \,r^2\,+\,{\mathcal {O}}(r^3)\right] \,, \end{aligned}$$

which holds for any $m\in (0,m_{\textrm{max}})$, provided that *u* is normalized so that $\max _M(u)=u_{\textrm{max}}(m)$. It is then not hard to compute the following expansion:

5.16 $$\begin{aligned} \frac{|\nabla u|^2}{\Psi ^2\left[ 1-(n-2)m\Psi ^{-n}\right] ^2}\,=\,1\,+\,\frac{2(n-1)}{3}\,\left( \frac{u_{\textrm{max}}(m)}{r_0(m)}-\frac{\textrm{H}}{n-1}\right) \,r\,+\,{\mathcal {O}}(r^2).\nonumber \\ \end{aligned}$$

By definition, *r* is positive in *A* and negative in *B*. Comparing with (5.15), the result follows at once. $\square$

We are now ready to prove Theorem 5.1 for the non-cylindrical case. Since *A* is outer, from Proposition 5.6 we get

$$\begin{aligned} \frac{\textrm{H}}{n-1}\,\geqq \,\frac{u_{\textrm{max}}(m_A)}{r_0(m_A)}, \end{aligned}$$

where $\textrm{H}$ is the mean curvature of $\Sigma$ with respect to the normal pointing inside *A*. In particular $\textrm{H}$ is positive everywhere on $\Sigma$. If *B* were also outer, then in the same way we would obtain that the mean curvature of $\Sigma$ with respect to the opposite normal (the one pointing inside *B*) is also positive and this would be a contradiction. Similarly, if *B* were cylindrical, then Proposition 5.5 would tell us that the mean curvature of $\Sigma$ with respect to the opposite normal is nonnegative, again leading to a contradiction. Therefore, *B* must be inner. Applying Proposition 5.6 to both *A* and *B*, we get

5.17 $$\begin{aligned} \frac{u_{\textrm{max}}(m_A)}{r_0(m_A)}\,\leqq \,\frac{\textrm{H}}{n-1}\,\leqq \,\frac{u_{\textrm{max}}(m_B)}{r_0(m_B)}. \end{aligned}$$

Since the function

$$\begin{aligned} m\mapsto \frac{u_{\textrm{max}}(m)}{r_0(m)}\,=\,\sqrt{\frac{1}{r_0^2(m)}\,-\,\frac{1}{r_0^2(m_{\textrm{max}})}}\,=\,\sqrt{\frac{1}{[(n-2)m]^{2/n}}-\frac{n}{n-2}} \end{aligned}$$

is clearly monotonically decreasing, necessarily we must have $m_A\geqq m_B$. Furthermore, if $m_A=m=m_B$, then formula (5.17) tells us that

5.18 $$\begin{aligned} \frac{\textrm{H}}{n-1}\,=\,\frac{u_{\textrm{max}}(m)}{r_0(m)}\,=\,\sqrt{\frac{1}{r_0^2(m)}\,-\,\frac{1}{r_0^2(m_{\textrm{max}})}}. \end{aligned}$$

Substituting this information inside the expansions for $\Psi$ and $|\nabla u|^2$, we can refine (5.16) and compute that, if (5.18) holds, then

$$\begin{aligned} \frac{|\nabla u|^2}{\Psi ^2\left[ 1-(n-2)m\Psi ^{-n}\right] ^2}\,=\,1\,+\,\frac{1}{2}\,|\mathring{\textrm{h}}|^2\,r^2\,+\,o(r^2). \end{aligned}$$

From (5.15) it follows then that $|\mathring{\textrm{h}}|=0$, that is,

5.19 $$\begin{aligned} \textrm{h}=\frac{\textrm{H}}{n-1}\,g^\Sigma \,=\,\sqrt{\frac{1}{r_0^2(m)}\,-\,\frac{1}{r_0^2(m_{\textrm{max}})}}\,g^\Sigma . \end{aligned}$$

We also know from (4.3) that $\textrm{Ric}(\nu ,\nu )=0$ on $\Sigma$. Substituting these pieces of information in the Gauss-Codazzi equation, we get

5.20 $$\begin{aligned} {\mathrm R}^\Sigma \,&=\,{\mathrm R}\,-\,2\,\textrm{Ric}(\nu ,\nu )\,+\,\textrm{H}^2\,-\,|\textrm{h}|^2\nonumber \\&=\,n(n-1)\,+\,\frac{(n-1)(n-2)}{r_0^2(m)}\,-\,\frac{(n-1)(n-2)}{r_0^2(m_{\textrm{max}})}\nonumber \\&=\,\frac{(n-1)(n-2)}{r_0^2(m)}\,. \end{aligned}$$

This concludes the proof.

### Uniqueness of the schwarzschild–de sitter black hole

We are now ready to prove Theorem 1.1, which we restate here for the reader’s convenience.

#### Theorem 5.7

Let (*M*, *g*, *u*) be a compact orientable 3-dimensional static solution with cosmological constant $\Lambda >0$. Assume that the set

$$\begin{aligned} \textrm{MAX}(u) \,=\,\{ x\in M:\,u(x)\,=\, u_\textrm{max}\} \end{aligned}$$

is disconnecting the manifold *M* into an outer region $M_+$ and an inner region $M_-$ having the same *virtual mass* $0< m < 1/(3\sqrt{3})$. Then (*M*, *g*) is isometric to the Schwarzschild–de Sitter solution with mass parameter *m*.

#### Proof

Applying Theorem 5.1-(*i*) with $A=M_+$, $B=M_-$ and $\Sigma =\overline{M_+}\cap \overline{M_-}$, we deduce that $\Sigma$ is totally umbilical and that the metric $g^\Sigma$ induced by $g$ on $\Sigma$ has constant positive scalar curvature equal to $2r_0^{-2}(m)$. Since $\Sigma$ is 2-dimensional, it follows that $\Sigma$ is isometric to a sphere of radius $r_0(m)$ (in particular, $g^\Sigma$ is Einstein).

We can then apply Theorem 1.2 to conclude that (*M*, *g*, *u*) is covered by a BK solution. Recall that the BK solution is a warped product of the form $[r_-,r_+]\times S$ with metric $dr^2/u^2 + r^2 g_{S}$, where $(S,g_S)$ is a simply connected 2-dimensional manifold with $\textrm{Ric}_{g_S}=g_S$ and $u^2=1-r^2-2\,m/r$. On the other hand, from the Uniformization Theorem we know that *S* is a 2-dimensional sphere, hence (*M*, *g*, *u*) must be a quotient of the Schwarzschild–de Sitter solution. The conclusion now follows easily from the fact that there are no nontrivial quotients of the sphere that preserve orientability. $\square$

Similarly, one can prove the analogous result for the Nariai solution, by employing Theorem 5.1-(*ii*) in place of Theorem 5.1-(*i*).

#### Theorem 5.8

Let (*M*, *g*, *u*) be a compact orientable 3-dimensional solution of the vacuum Einstein equations with cosmological constant $\Lambda >0$. Assume that the set

$$\begin{aligned} \textrm{MAX}(u) \,=\,\{ x\in M:\,u(x)\,=\, u_\textrm{max}\} \end{aligned}$$

is disconnecting the manifold *M* into two regions having the same “virtual mass” $m= 1/(3\sqrt{3})$. Then (*M*, *g*) is isometric to the Nariai solution.

As mentioned in the Introduction, Theorems 5.7 and 5.8 improve on the uniqueness result for the Schwarzschild–de Sitter solution proposed in [12, Theorem 7.4]. Let us briefly recall the strategy in [12]. In [12], a monotonicity argument – inspired by [3], where the case $\Lambda =0$ was considered – was employed to deduce the inequality

$$\begin{aligned} r_+^2(m)|\Sigma |\leqq r_0^2(m)|\partial M_+| \end{aligned}$$

between the areas of the interface and of the outer boundary. The 3-dimensional version of Theorem 5.1, and in particular the identity ${\mathrm R}^\Sigma =2/r_0^2(m)$, was then exploited together with the Gauss–Bonnet formula to deduce

$$\begin{aligned} |\Sigma |=\frac{r_0^2(m)}{2}\int _\Sigma {\mathrm R}^\Sigma d\sigma =4\pi r_0^2(m). \end{aligned}$$

Finally, assuming connectedness of $\partial M_+$, the sharp and rigid Riemannian Penrose inequality

$$\begin{aligned} |\partial M_+|\leqq 4\pi r_+^2(m) \end{aligned}$$

was established in the same paper (see [12, Theorem 1.4]). It was then enough to put together these inequalities, to deduce that in fact they were all saturated, easily concluding the proof by rigidity.

A couple of comments are in order. First of all, this proof is markedly three-dimensional as it relies heavily on the Gauss–Bonnet Theorem. Secondly, the global nature of the proof makes it so that it is necessary to add the undesirable assumption of connectedness of the outer boundary $\partial M_+$.

In contrast, the proof proposed in this paper is more local in nature, as it is based only on an analysis in a neighborhood of the interface. In particular, the horizons do not play any role and there is no need to require connectedness of $\partial M_+$. Furthermore, the proposed proof does not rely on the Gauss–Bonnet formula, but only on Theorem 5.1 and Theorem 1.2. Since both these results work in any dimension $n\geqq 3$, one may wonder whether it is possible to prove a higher-dimensional version of Theorem 5.7 as well.

Unfortunately the route towards such an extension seems not so easy to find, and it is even impossible from a local perspective. Indeed, we observe that the thesis of Theorem 5.1 says that $\Sigma$ is CMC umbilical with constant scalar curvature. In order to be able to invoke Theorem 1.2, one would need to further argue that this forces the metric induced on $\Sigma$ to be Einstein. In the next section we show that in general this is not the case, for dimensions $n\geqq 4$, if the strategy only relies on local arguments. In Proposition 6.1 we will show that for any compact, real analytic manifold $\Sigma$ and for any choice of real analytic symmetric 2-tensors $g^{\top },h$ on $\Sigma$, we can find a local solution to the static vacuum Einstein equations on $\Sigma \times (-\varepsilon ,\varepsilon )$ such that the metric induced on $\Sigma =\Sigma \times \{0\}$ is $g^{\top }$ and the second fundamental form of $\Sigma$ is *h*. Hence, in dimension $n\geqq 4$ one can clearly choose $g^{\top }$ and *h* in such a way that the thesis of Theorem 5.1 is satisfied (i.e., $\Sigma$ is CMC umbilical – it is enough to take $h=constant\cdot g^{\top }$ – and the metric $g^{\top }$ induced on $\Sigma$ has constant scalar curvature), but the solution does not belong to the BK family, as long as $g^{\top }$ is not Einstein.

## Local Solutions

A triple (*M*, *g*, *u*) will be said to satisfy the Riemannian static Einstein equations if the spacetime metric

$$\begin{aligned} \gamma _{\text{ Lor }}: = -u^2 dt^2 + g , \quad \partial _t u = 0 = \partial _t g \, \end{aligned}$$

satisfies the vacuum Einstein equations, possibly with a cosmological constant. In this section we will outline how to construct such real-analytic triples (*M*, *g*, *u*), by mimicking the Cauchy problem in general relativity, invoking the Cauchy-Kovalevskaya theorem to justify existence. The construction is a straightforward adaptation of that of Darmois [23], a summary of the argument can be found in [16].

We note that the Cauchy-Kovalevskaya theorem, in the current setting, does not care about the signature of the metric, and that the equations for an Einstein Riemannian metric

$$\begin{aligned} \gamma _{\text{ Riem }}: = u^2 dt^2 + g , \quad \partial _t u = 0 = \partial _t g, \end{aligned}$$

are identical to the Lorentzian ones. There is, however a slight advantage of solving the Riemannian equations instead, as then zeros of *u* can be smoothly mapped to axes of rotation for the metric $\gamma _{\text{ Riem }}$, avoiding the problem of equations which are potentially singular at the zeros of *u*. We will, however, not pursue this line of thought, as our main interest here is to solve the equations starting from a hypersurface on which *u* is nowhere vanishing.

Thus, we seek to construct a solution of the static Einstein equations of the form

$$\begin{aligned} g= dr^2 + {g{}^{\!\top }}(r) , \end{aligned}$$

where ${g{}^{\!\top }}(r)$ is an *r*-dependent family of metrics on a, say compact, real analytic manifold $\Sigma$. The initial data at $r=0$ are *u*(0), ${g{}^{\!\top }}(0)$ and their first *r*-derivatives at $r=0$, all taken to be real-analytic. The Cauchy-Kovalevskaya theorem in Gauss coordinates, as in Theorem 4.2, provides existence of an interval $r\in (-r_0,r_0)$ and a metric

$$\begin{aligned} \gamma _{\text{ Lor }} = dr^2 \underbrace{-u^2 dt^2 + {g{}^{\!\top }}(r)}_{=:{\hat{g}}(r)} , \end{aligned}$$

on $\{r\in (-r_0,r_0),t\in {\mathbb {R}}\}\times \Sigma$. The metric $\gamma _{\text{ Lor }}$ will be *t*-independent and will satisfy the vacuum Einstein equations with a cosmological constant provided that the initial data fields

$$\begin{aligned} {\hat{g}}|_{r=0}:= -u(0)^2 dt^2 + {g{}^{\!\top }}(0) \quad \text{ and } \quad \partial _r {\hat{g}}|_{r=0} \end{aligned}$$

are chosen to be time-independent and satisfy the usual general relativistic constraint equations. Summarizing, the Cauchy–Kovalevskaya Theorem in the context of static spacetimes gives the following:

### Proposition 6.1

Given a compact $(n-1)$-dimensional real analytic Riemannian manifold $(\Sigma ,g^\Sigma )$ and real analytic functions *v*, *w*, *h* on $\Sigma$ with $v>0$, there exists a static spacetime $X={\mathbb {R}}\times (-r_0,r_0)\times \Sigma$ with spacetime metric

$$\begin{aligned} \gamma _{\text{ Lor }}: = -u^2 dt^2+dr^2 + g^\top (r), \end{aligned}$$

where $g^\top$ is a collection of metrics on $\Sigma$ with $g^\top (0)=g^\Sigma$, $\partial g^\top /\partial r_{|_{\Sigma }}=h$, $u_{|_\Sigma }=v$ and $\partial u/\partial r_{|_{\Sigma }}=w$.

The above provides many new local solutions of the static Einstein equations. Here local refers to the fact, that the solutions might not necessarily extend to boundaries on which *u* vanishes.

The question then arises, which data on $\Sigma$ leads to manifolds *M* which are bounded by Killing horizons; equivalently, manifolds with boundary on which *u* vanishes. When starting from a critical level set of *u*, a sufficient condition for this is provided by Theorem 1.2.

## Appendix A Gradient estimate

In this appendix we show the equivalence between the elliptic inequality (5.5) and [12, Formula (3.24)]. To this end, we start by recovering some useful notation and formulas from [12]. First of all, we denote by $\psi :[0,u_{\textrm{max}}]\rightarrow {\mathbb {R}}$ the function such that $\Psi =\psi \circ u$. The derivative ${\dot{\psi }}$ of $\psi$ satisfies

A.1 $$\begin{aligned} {\dot{\psi }}(u)=-\frac{u\Psi ^{n-1}}{\Psi ^n-(n-2)m}\quad \hbox {and}\quad |\nabla u_m|_{g_m}=\frac{u}{|{\dot{\psi }}(u)|}. \end{aligned}$$

The differential inequality in [12, Formula (3.24)] is written in terms of

$$\begin{aligned} w=u\frac{\Psi }{|{\dot{\psi }}(u)|}\left( 1-|\textrm{d}\varphi |_{\tilde{g}}^2\right) , \end{aligned}$$

where ${\tilde{g}}=g/\Psi ^2$ and $\varphi$ is the *pseudo-affine function*. We will not need the precise definition of $\varphi$ but we will need the following relations involving $\textrm{d}\varphi$, again proved in [12]:

$$\begin{aligned} \textrm{d}\varphi =-\frac{{\dot{\psi }}(u)}{u\Psi }\textrm{d}u,\qquad |\textrm{d}\varphi |_{{\tilde{g}}}=\frac{|{\dot{\psi }}(u)|}{u}|\nabla u|=\frac{|\nabla u|}{|\nabla u_m|_{g_m}}. \end{aligned}$$

It is now easy to rewrite the quantity *w* above as

$$\begin{aligned} w=\Psi |\nabla u_m|_{g_m}\left( 1-\frac{|\nabla u|^2}{|\nabla u_m|_{g_m}^2}\right) =\frac{\Psi }{|\nabla u_m|_{g_m}}\left( |\nabla u_m|_{g_m}^2-|\nabla u|^2\right) . \end{aligned}$$

In particular, *w* coincide with the function *W* defined in (5.4) above. Starting from [12, Formula (3.24)], we then compute

$$\begin{aligned} \Delta _{\tilde{g}}W\,&\leqq \,\left[ (n-2)u-\frac{\Psi }{{\dot{\psi }}(u)}\right] \langle \textrm{d}W\,|\,\textrm{d}\varphi \rangle _{\tilde{g}}\\ {}&\quad +n(n-2)m\Psi ^{2-n}{\dot{\psi }}^2(u)\left[ (n-2)+(n+2)|\textrm{d}\varphi |_{\tilde{g}^2}\right] W\\&=\left[ -(n-2)\frac{{\dot{\psi }}(u)}{\Psi }+\frac{1}{u}\right] \langle \textrm{d}W\,|\,\textrm{d}u\rangle _{{\tilde{g}}}\\ {}&\quad + \frac{n(n-2)mu^2\Psi ^n}{\left[ \Psi ^n-(n-2)m\right] ^2}\left[ (n-2)+(n+2)\frac{|\nabla u|^2}{|\nabla u_m|^2_{g_m}}\right] W\,. \end{aligned}$$

Using (A.1) to substitute ${\dot{\psi }}(u)$ in the first square bracket, we obtain precisely (5.5), as desired.
